# Functionalized hydrocaffeic acid-chitosan/EGTA hydrogel rescues mitochondrial dysfunction for immunomodulation and joint repair in rheumatoid arthritis

**DOI:** 10.1016/j.mtbio.2025.102605

**Published:** 2025-11-28

**Authors:** Jianxin Li, Yingchen Ni, Yanjie Tao, Hao Cai, Anqi Wu, Fengyuan Zhang, Shijie Meng, Yuyang Luo, Weidong Zhang, Youhua Wang

**Affiliations:** a Department of Orthopaedic Surgery, Affiliated Hospital of Nantong University, Nantong, 226001, China; b Affiliated Hospital of Nantong University, Medical School of Nantong University, Nantong, 226001, China; c Department of Laboratory Medicine, Nantong First People's Hospital, The Second Affiliated Hospital of Nantong University, Nantong, 226001, China

**Keywords:** Rheumatoid arthritis, Mitochondrial dysfunction, Calcium overload, Reactive oxygen species, Cellular senescence, Cartilage regeneration

## Abstract

Rheumatoid arthritis (RA) is a chronic autoimmune disorder marked by progressive joint degradation, with mitochondrial dysfunction significantly contributing to its pathogenesis. Despite extensive research into therapeutic strategies, successfully addressing mitochondrial dysfunction in RA poses a significant challenge. This paper presents an innovative functionalization method, deploying hydrocaffeic acid-modified chitosan, in conjunction with the selective calcium chelator ethylene glycol bis(β-aminoethyl ether)-N,N,N’,N’-tetraacetic acid (EGTA), and incorporating stromal cell-derived factor 1 alpha (SDF-1α). *In vitro*, this functionalized hydrogel exhibited a significant decrease in intracellular reactive oxygen species (ROS) levels, stabilization of mitochondrial membrane potential, mitigation of calcium overload, inhibition of mitochondrial dysfunction-induced cellular senescence, and a reduction in the release of senescence-associated secretory phenotype components. *In vivo*, this hydrogel effectively modulated immune responses and aided cartilage repair in a collagen-induced arthritis rat model. From a mechanistic perspective, high-throughput sequencing suggests that the therapeutic efficacy of this hydrogel may be associated with its ability to modulate mitochondrial function and inflammatory pathways. In summary, the hydrocaffeic acid- and EGTA-based functionalization strategy provides an innovative and straightforward process for integrating multiple functionalities into a single delivery platform, demonstrating the potential for tissue regeneration applications extending beyond RA.

## 1 Introduction

Rheumatoid arthritis (RA) is a complex autoimmune disease largely characterized by enduring inflammation of the synovial membrane, the progressive deterioration of articular cartilage and bone, and systemic inflammatory responses [1,2]. Despite remarkable advancements in understanding the underlying mechanisms, the pathogenesis of RA is still not fully comprehended. Increasing evidence underscores mitochondrial dysfunction as a pivotal factor propelling the progression of this disease [3]. As the powerhouse of the cell, mitochondria perform a crucial function in maintaining cellular energy metabolism, redox homeostasis, and calcium ion equilibrium [4]. Thus, examining mitochondrial dysfunction and its role in the progression of RA is critical for decoding the disease's pathogenesis and progressing the creation of targeted therapeutic interventions.

Mitochondrial dysfunction is a complex process influenced by various interrelated factors, including calcium ion dysregulation, excessive production of reactive oxygen species (ROS), and persistent inflammation [5,6]. These elements collectively compromise mitochondrial integrity and heighten pathological states. Excessive calcium accumulation, in particular, serves a crucial role in propelling mitochondrial dysfunction and speeding up the pathological advancement of RA through a variety of mechanisms [7,8]. Under conditions of decreased mitochondrial membrane potential, the ability of mitochondria to sequester calcium is notably reduced. This dysfunction can lead to the release of stored calcium ions, resulting in abnormally high cytosolic calcium levels [9]. The consequent calcium overload activates several calcium-dependent enzymes, such as calcineurin and calpains, which damage the cytoskeleton and membrane structure, while also intensifying oxidative stress by modifying metabolic pathways. At the same time, calcium imbalance disrupts the mitochondrial electron transport chain, leading to electron leakage and an overproduction of ROS [10,11]. ROS accumulation directly harms mondrial DNA, proteins, and membrane lipids and triggers pro-inflammatory signaling pathways, including Nuclear Factor Kappa-B (NF-κB) and the NOD-like receptor family pyrin domain-containing protein 3 (NLRP3) inflammasome, through oxidative stress [12,13]. This positive feedback loop of ROS production further amplifies mitochondrial dysfunction, perpetuating a harmful cycle. On the whole, the complex interaction between calcium dysregulation, oxidative stress, and persistent inflammation establishes a self-perpetuating cycle of mitochondrial dysfunction, ultimately propelling the progression of RA and intensifying joint damage.

Cellular senescence, initially identified by Hayflick et al. is recognized as a permanent state characterized by cell cycle arrest and is associated with various pathological conditions [14]. Recent evidence convincingly demonstrates that the accumulation of senescent cells stands as a hallmark of aging across multiple species [15,16]. A substantial body of literature suggests that the onset of senescence is primarily driven by the secretion of inflammatory molecules that impair the function of neighboring cells. Many pro-inflammatory cytokines, including Interleukin-6 (IL-6) and interleukin-8 (IL-8), have been closely linked with stress-induced premature senescence [17]. In this context, mitochondrial dysfunction is recognized as a critical driver of cellular senescence. Notably, dynamin-related protein 1 (Drp1), a key mitochondrial fission protein, when abnormally activated, leads to excessive mitochondrial fragmentation and increased ROS production, thereby amplifying inflammatory responses and accelerating cellular senescence processes [18]. As mentioned in the literature, Drp1 is involved in the migration of fibroblast-like synoviocytes and CD4^+^ T cells, which exacerbates RA inflammation [19,20]. Accordingly, the dysregulation of Drp1 is associated not only with cellular senescence and reduced lifespan but also with RA progression, highlighting the essential role of mitochondrial dynamics in joint inflammation.

The contemporary management of RA primarily uses anti-inflammatory and immunomodulatory strategies, encompassing nonsteroidal anti-inflammatory drugs (NSAIDs), glucocorticoids, disease-modifying antirheumatic drugs, and biological agents such as Tumor Necrosis Factor-alpha (TNF-α) inhibitors and IL-6 receptor antagonists [1,2]. These therapeutic methods alleviate symptoms and slow down disease progression, however, their effectiveness is limited, they may cause adverse side effects, and drug resistance can develop. Furthermore, these treatments do not facilitate the regeneration of the joint microenvironment or the healing of cartilage, underscoring the crucial need for precise and effective strategies aimed at enhancing drug stability, targeting specificity, and improving therapeutic outcomes. To meet this challenge, biomaterial-based delivery platforms offer a promising solution for treating RA by enabling the targeted delivery of therapeutic agents, improving bioavailability, minimizing side effects, and enhancing therapeutic efficacy. Chitosan (CS), a natural polymer known for its biocompatibility and anti-inflammatory properties, can form a cross-linked network ideal for loading and delivering bioactive molecules, such as antioxidants and anti-inflammatory agents [21,22], thereby making it an excellent material for RA treatment.

**Scheme 1:**
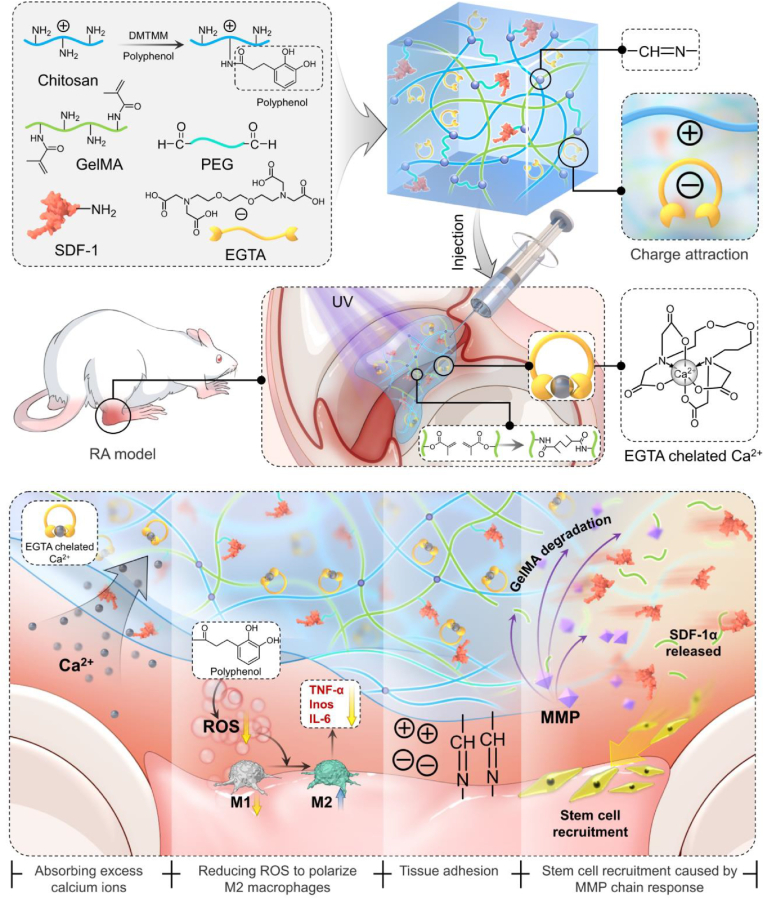
Schematic illustration of the design and preparation of a composite hydrogel containing EGTA-coated chitosan loading SDF-1α for the treatment of RA which can eliminate ROS and promote macrophage M2 polarization. Composite hydrogel slowly degrade and constantly release EGTA and SDF-1α, which can regulate immune response and cartilage formation.

In this study, we developed a nanocomposite hydrogel by combining CS with gelatin methacrylate (GelMA, which exhibits matrix metalloproteinase (MMP)-responsive degradation), and polyethylene glycol (PEG). This bio-inspired network exhibits multifunctionality, including enhanced adhesiveness, injectability, MMP-responsive degradability, compatibility, and the capacity to restore mitochondrial function. Specifically, hydrocaffeic acid was grafted onto the primary amino groups using 4-(4,6-Dimethoxy-1,3,5-triazin-2-yl)-4-methylmorpholinium chloride (DMTMM), while ethylene glycol bis-(β-aminoethylether)-N,N,N′,N′-tetraacetic acid (EGTA) was incorporated into the PEG network to create an EGTA-based anti-inflammatory hydrogel platform (Scheme 1). Hydrocaffeic acid (3,4-dihydroxyphenylacetic acid), a phenolic compound structurally related to caffeic acid, exhibits strong antioxidant capacity through scavenging ROS, and previous studies have demonstrated that hydrocaffeic acid together with related dihydroxyphenolic acids can mitigate oxidative stress while simultaneously attenuating inflammatory responses during tissue injury [[23], [24], [25]]. At the same time, EGTA exhibits a selective chelation affinity for calcium ions (Ca^2+^), consequently mitigating calcium-induced toxicity and preserving mitochondrial integrity. By addressing both calcium overload and ROS accumulation, this composite material sustains mitochondrial function and improves cellular energy metabolism, reduces inflammatory damage, and delays cellular aging.

Cartilage progenitor cell (CPC)-based therapy is currently being explored in our laboratory as a potential strategy for repairing degenerated articular cartilage and promoting regeneration in RA. However, the therapeutic efficacy can often be undermined by the inflammatory environment within the joint cavity. To address this issue, we devised an injectable adhesive hydrogel that responds to MMP. This hydrogel serves as a composite drug delivery system, integrating EGTA, CS loaded with hydrocaffeic acid, and stromal cell-derived factor-1 alpha (SDF-1α)-coupled GelMA. Thanks to its multifunctional characteristics, the hydrogel specifically regulates Ca^2+^ levels, diminishes ROS, preserves mitochondrial integrity, sequentially recruits cells *in situ*, and effectively promotes cartilage regeneration.

## 2 Methods and materials

### 2.1 Patients and control individuals

Synovial fluid was collected from 20 RA patients and 20 osteoarthritis (OA) patients who visited the Department of Orthopedics, Affiliated Hospital of Nantong University. All patients included in the study were diagnosed clinically. The synovial fluid was obtained from the knee joints of RA patients and OA patients who underwent total knee replacement. The study was approved by the Ethics Committee of the Affiliated Hospital of Nantong University (2020-L136) and conducted in accordance with the Declaration of Helsinki.

### 2.2 Preparation of materials

100 mg chitosan (Sigma-Aldrich, USA), 50 mg hydrocaffeic acid (FEIYUBIO, China) and 74 mg DMTMM (Sigma-Aldrich, USA) were dissolved in 2-(N-Morpholino) ethanesulfonic acid (MES, Sigma-Aldrich, USA) solution. The resulting product is stored in nitrogen at room temperature (RT) for 12 h (h). After further dialysis treatment, it was placed at −80 °C, then freeze-dried in refrigerator and stored at −20 °C. An appropriate quantity of PEG (Sigma-Aldrich, USA) should be accurately weighed and subsequently dissolved in phosphate-buffered saline (PBS, HyClone, USA) to formulate a 2 % PEG hydrogel solution. To prepare the mixed hydrogel prepolymer solution, combine hydrogel solutions of 1 % chitosan, 2 % PEG, and 5 % GelMA (EFL, China) in a volume ratio of 2:1:1. Subsequently, Chitosan-CAL/GelMA /PEG-Ald were mixed and illuminated with ultraviolet light (with a wavelength of 405 nm). The hydrogel system comprises multiple functional components, whose abbreviations and full chemical names are summarized in Fig. S1 for reference. This nomenclature system will be used consistently throughout the manuscript.

### 2.3 Characterization of materials

The surface morphologies of different components of hydrogels were examined by field-emission scanning electron microscope (SEM, FEI, USA). The chemical composition of the samples was determined by Fourier-transform infrared spectroscopy (FTIR, Nicolet iS 10, Thermo Fisher Scientific, USA). The spectral characteristics of the caffeine acid-functionalized chitosan (CS-CAL) were analyzed using UV–vis spectrophotometry (BIO-DL, China). Its molecular structure was further characterized by proton nuclear magnetic resonance (^1^H NMR, 600 MHz, Bruker, Germany) with D_2_O as the solvent. The rheological properties of the hydrogels were tested with a Discovery HR2 Hybrid Rheometer (TA Instrument, USA). The hydrogels’ moduli were measured over strain amplitudes of 0.01–1000 % and frequencies of 0.01–100 Hz. Rheological characterization via continuous flow measurements assessed the shear-thinning properties by monitoring viscosity as a function of applied shear stress.

### 2.4 Adhesion and tensile tests

The macroscopic adhesion of hydrogels was evaluated using a universal testing machine (Instron, USA). Hydrogel samples were sandwiched between two stainless-steel plates and subjected to vertical stretching at a constant crosshead speed of 10 mm/min until detachment or rupture occurred. During the process, the deformation and filament extension of the hydrogel were recorded by a digital camera to visualize its viscoelastic dissipation and adhesive performance.

### 2.5 *In vitro* drug release study

MMP13 was used to simulate the microenvironment *in vivo*. The CSE-CAL@GS hydrogels were incubated at 37 °C in two groups (PBS and MMP13). The supernatants were collected on days (d) 1, 3, 5, 7, 9, 11, 14, and 21. The release of SDF-1α was detected using an SDF-1α ELISA kit (Beyotime, China). Additionally, the release of EGTA was indirectly measured using a calcium ion detection kit (Beyotime, China).

### 2.6 Isolation of rat cartilage stem cells

Articular cartilage of knee joint was harvested from 5-day-old red-skinned rats anesthetized with 2 % isoflurane (RWD life science, China). The collected articular cartilage tissues were minced into small fragments, washed in PBS containing penicillin-streptomycin (Pen-Strep), inverted to mix, and centrifuged at 1000 rpm for 5 min (repeated 3 times). After discarding the supernatant, 12 mL of trypsin was added to the centrifuge tube and digested on a thermostatic shaker (37 °C, 101 rpm, 10 min). The tissues were then washed twice with Pen-Strep-supplemented PBS. Subsequently, 2 mL of 0.2 % type II collagenase (Biosharp, China) was added and digested on the shaker for 6–8 h. The digested solution was filtered through a cell strainer, aliquoted into centrifuge tubes, and centrifuged at 1500 rpm for 5 min. Finally, the supernatant was discarded, and the pellet was resuspended in culture medium.

### 2.7 Cell culture

Nutrient Mixture F12 (Gibco, USA) with 1 % Pen-Strep and 10 % fetal bovine serum (Gibco, USA) was used for cell culture. The cells were incubated in an incubator at 37 °C with 5 % CO_2_, and CPCs at early passages (passages 3–5) were used for subsequent experiments. DMEM with 1 % Pen-Strep and 10 % fetal bovine serum was used for RAW 264.7 cell line culture.

### 2.8 Live/Dead staining assay

The cell viability of different GelMA microspheres was measured by Live/Dead cell staining kit (Dojindo Kagaku, Japan), in which the live cells were stained in green while the dead cells were stained in red under fluorescence microscope (ZEISS, Axio vert5, Germany). The CPCs with a density of 2 × 10^4^ cells/mL were cultured in 24-well plates at 37 °C and 5 % CO_2_ atmosphere, and the culture medium was changed every two days. Subsequently, the CPCs were cocultured with CS-CAL@GS, CSE-CAL@GS, CS-CAL@GS or CSE-CAL@GS solutions 5 d in triplicate. Finally, the cells were stained with the Live/Dead cell staining dye (200 μL) for 15 min, and cell morphology was investigated by the fluorescence microscopy.

### 2.9 Cytoskeleton staining

To investigate the biocompatibility of different composite hydrogels, CPCs were seeded on the surface of composite hydrogels (CS-CAL@GS, CSE-CAL@GS, CS-CAL@GS or CSE-CAL@GS at a density of 5 × 10^4^ per well. After 3 days, CPCs were washed with PBS and fixed with 4 % paraformaldehyde. The CPCs were further perforated with 0.3 % TritonX-100. Then, 3 % bovine serum albumin solution was added, and the samples were kept at 4 °C overnight. CPCs were then incubated with phalloidin and subsequently stained with 4′,6-diamidino-2-phenylindole. Images were acquired using an inverted fluorescence microscope (ZEISS, Axio vert5, Germany).

### 2.10 Cytotoxicity experiment

The CPCs and RAW264.7 were cultured and co-cultured with the composite hydrogel similarly as before. The Cell Counting Kit-8 (CCK-8, Beyotime, China) assay was used to evaluate cell cytotoxicity and cell proliferation at different time points (1, 4 and 7 d). Briefly, 20 μL of CCK-8 solution and 200 μL of fresh medium were added into each well, and the plates were incubated at 37 °C for another 2 h. Finally, the mixed solution was transferred to the 96-well plates in darkness, and the absorbance was measured through a microplate reader (Thermo Fisher, MK3, Switzerland) at 450 nm.

### 2.11 Intracellular ROS measurement

ROS Scavenging Potential Evaluation: According to the assay kit manufacturer's instruction, 2″,7″-Dichlorodihydroflfluorescein diacetate (H2DCFH-DA, Biosharp, China) staining, JC-1 assays (MedChemExpress, USA).

### 2.12 Fluo-4 calcium assay

According to the instructions, the cells were washed with PBS and then added with Fluo-4 staining (Beyotime, China) solution and incubated at 37 °C in the dark for 30 min. Finally, a fluorescence microscope (ZEISS, Axio vert5, Germany) is used to observe the fluorescence intensity or a flow cytometer is used for detection.

### 2.13 Chemotactic effect of SDF-1α on CPCs

2 × 10^4^ cells were seeded in the upper chamber of Transwell inserts. Complete medium containing SDF-1α (Peprotech, USA), CSE-CAL@GS or no chemokines was placed in the lower chamber. After 24 h, the cells in the upper chamber were wiped off with cotton swabs and the cells in the lower chamber were fixed with 4 % paraformaldehyde (PFA) for 30 min and stained with 0.2 % crystal violet (MedChemExpress, USA) for 15 min. Migrated cells were observed microscopically and cell numbers were quantified by counting. For the wound healing assay, cells were seeded in a 6-well plate. A wound was made in the center of each well by scratching with a 200 μl pipette tip. Then, the cells were cultured in the same groups as mentioned above. Scratch wounds were imaged using an inverted microscope (Leica, USA) at 0, 12 and 24 h post-wounding, and the migration areas were measured using ImageJ software (National Institutes of Health, MD, USA).

### 2.14 Total RNA extraction and cDNA synthesis and quantitative real time polymerase chain reaction (qRT–PCR) analysis

CPCs and RAW264.7 at a density of 2 × 10^5^ cells/mL were cultured in 6-well plates and treated with lipopolysaccharide (LPS, MedChemExpress, USA) (1ug/ml)+2.5 mM CaCl_2_ (Sigma-Aldrich, USA) for 24 h. The cells were co-cultured with the hydrogel for 5 d. The CPCs and RAW264.7 were washed twice with PBS, the total RNA of CPCs was extracted with TRIzol reagent (Invitrogen, USA), and the purity and concentration of the RNA solution were obtained by measuring the relative absorbance at 280 nm and 260 nm wavelengths. Then, cDNA was synthesized using a synthesis kit (Vazyme, China). QuantStudio®5 real-time fluorescent quantitative PCR system was used for qRT-PCR detection. GADPH serves as an internal reference. Primer sequences are listed in Table 1 (Sangon Biotech, China).

**Table 1:** Primers for qPCR.

Gene	Forward (5′-3′)	Reverse (5′-3′)
***Ptgs2***	TCCTGTGGTGCTGGCTGTG	GAGATGGGCTGTTGTGTCATACTG
***Sod1***	TGACTTGGGCAAAGGTGGAAATG	CAGTTTAGCAGGACAGCAGATGAG
***TNFA***	AAAGGACACCATGAGCACGGAAAG	CGCCACGAGCAGGAATGAGAAG
***IL6***	ACTTCCAGCCAGTTGCCTTCTTG	TGGTCTGTTGTGGGTGGTATCCTC
***Sod2***	GGCTAAGGATGGATGGAGTGGTAG	TCCGAATTAACAGTTGTCAGTCAGG
***Gapdh***	GACATGCCGCCTGGAGAAAC	AGCCCAGGATGCCCTTTAGT

### 2.15 Western immunoblotting

The Total Protein Extraction Kit was used to extract protein from cells. BCA protein assay kit (Beyotime, China) was used to measure the concentration of cellular proteins, and the proteins were separated by sodium dodecyl SDS polyacrylamide gel electrophoresis (SDS-PAGE, EpiZyme, China) electrophoresis and transferred to polyvinylidene fluoride (PVDF, Beyotime, China) membrane. After blocking with 5 % skim milk for 2 h, the membrane was incubated with primary antibody overnight at 4 °C. Then the membrane was incubated with the corresponding secondary antibody for 2 h at RT after washing three times with TBST. Thereafter, protein bands were detected with a high-sensitivity ECL chemiluminescence kit (Vazyme, China). Finally, ImageJ software (NIH, Bethesda, MD, USA) was used for quantitative analysis. Primary antibodies chosen for this study are shown in Table 2.

**Table 2:** Primary antibodies used in this study.

Antibody	Vendor	Catalog number
**Drp1**	Abcam, Cambridge, UK	ab184247
**COX4**	Abcam, Cambridge, UK	ab202554
**NLRP3**	Proteintech, Wuhan, China	19771-1-AP
**Collagen II**	Abcam, Cambridge, UK	ab34712
**TNF-α**	Proteintech, Wuhan, China	17590-1-AP
**p-38**	Cell Signaling Technology, MA, USA	#9212
**p-p38**	Cell Signaling Technology, MA, USA	#4511
**Actin**	Proteintech, Wuhan, China	20536-1-AP
**p-Drp1**	Abcam, Cambridge, UK	ab314755
**IP3R**	Absin, Shanghai, China	abs148963

### 2.16 Enzyme-linked immunosorbent assay (ELISA)

TNF-α and IL-10 concentrations in cell culture supernatants were quantified using ELISA kits (Mlbio, China) according to the manufacturer's instructions. After 24 h of treatment with LPS (1 μg/mL) and CaCl_2_ (2.5 mM) in the presence or absence of CS-CAL@GS or CSE-CAL@GS, supernatants were collected for analysis. Absorbance at 450 nm (A450) was recorded with a microplate reader, and cytokine concentrations were calculated from standard curves.

### 2.17 Alcian blue staining

The cells were first stimulated with LPS (1 μg/mL) and CaCl_2_ (2.5 mM), then incubated for 24 h with either CS-CAL@GS or CSE-CAL@GS hydrogels. To evaluate the potential of hydrogels to promote cartilage matrix synthesis, cells were stained with Alcian Blue solution (Beyotime, China) at RT for 30 min. Images were acquired under a microscope (OLYMPUS, USA).

### 2.18 Senescence-associated β-galactosidase activity assay (SA-β-gal)

CPCs were first treated with LPS (1 μg/mL) and CaCl_2_ (2.5 mM) to establish an inflammatory model. After 24 h culture with either CSE-CAL@GS or CS-CAL@GS hydrogels, cells were PBS-washed, fixed (4 % PFA, 15 min), and then stained with SA-β-gal solution (Solarbio Biotechnology, China) for 15 min at RT. Senescent cells were quantified by counting blue-stained cells in 5 random microscope fields (OLYMPUS, USA).

### 2.19 Flow cytometry

RAW264.7 cells were seeded on composite hydrogels at a density of 2 × 10^4^ /mL. Cells were collected and labeled with antibodies against F4/80 (Biolegend, USA), CD86 (Biolegend, USA), and CD206 (Invitrogen, USA) after treatment with 1 μg/mL LPS and 2.5 mM CaCl_2_ for 24 h. CD86^+^/F4/80^+^ macrophages or CD206^+^/F4/80^+^ macrophages were identified after washing with PBS using a flow cytometer (BD LSRFortessa, USA). Additionally, ROS levels were measured by flow cytometry using the H2DCFH-DA kit (Biosharp, China) with 10 μM probe loading for 30 min at 37 °C.

### 2.20 Immunofluorescence staining

RAW264.7 cells at a density of 5000 cells/mL were cultured in 24-well plates with sterile coverslips, treated with LPS (1ug/ml) and 2.5 mM CaCl_2_ for 24 h, and then co-cultured with the above composite hydrogels. After 24 h of co-culture, RAW264.7 cells were fixed with 4 % PFA for 30 min, then permeabilized with 0.1 % Triton X-100 (Aladdin Biochemical Technology Co., Ltd., China), and treated with 3 % bovine serum albumin/phosphate Buffer (Sigma-Aldrich, USA) blocked at 25 °C. Cells were stained with a specific rabbit primary antibody against CD86 (1:200, Proteintech, China) and CD206 (1:200, Proteintech, China) and incubated overnight at 4 °C. Subsequently, cells were gently washed with PBS and reacted with a specific Alexa Fluor-conjugated secondary antibody in the dark for 1 h. Additionally, nuclei were counterstained with mounting medium containing 4,6-diamidino-2-phenylindole dilactic acid (DAPI, Beyotime, China). Finally, a fluorescence microscope is used to take images to observe the staining (OLYMPUS, USA).

### 2.21 Establishment and treatment of collagen-induced arthritis (CIA) model

Male wista rats (6–8 weeks) were purchased from Laboratory Animal Center Of Nantong University. All experimental procedures were performed according to the protocols approved by Laboratory Animal Center Of Nantong University (No. S20200323-124). The collagen type II (Chondrex, USA) was mixed with CFA (Chondrex, USA) to get the emulsifified solution (1/1, v/v). Then 200 μl of emulsifified solution was injected into 6-week-old Male wista/per rat through base of the tail. After 21 d, same emulsifified solution was again injected into the male wista/per rat for immune boosting. 28 d after the primary injection, materials were injected into the ankle joint of the rat. CPCs were injected biweekly.

### 2.22 Visualized monitoring of drug retention in articular cavity

6–8 weeks old male wista rats were used to assess the retention of CSE-CAL@GS. A single 100 μL injection of alkene-coupled fluorescent hydrogel dye combined with CSE-CAL@GS was administered into the articular cavity. Fluorescence was monitored immediately post-injection using an IVIS Spectrum system (PerkinElmer, USA) at excitation/emission wavelengths of 450/525 nm. Subsequent fluorescence measurements were conducted weekly for 4 w without additional drug administration.

### 2.23 Immunofluorescence and histomorphometry analysis

Following 4 weeks of treatment, the ankle joints of rats were excised and fixed with 10 % paraformaldehyde for 24 h. After this, tissues were decalcified, dehydration, embedded in paraffin, sliced, and stained with Hematoxylin & Eosin (H&E), Safranin O-Fast green (SO/FG) and Masson staining. Additional sections were used for fluorescence staining. The sections were initially blocked with 10 % (v/v) normal serum-blocking solution containing 1 % (w/v) BSA and 0.3 % (v/v) Triton X-100 for 2 h at RT. Next, sections were incubated with anti-CD86 (1:200, Proteintech, China), anti-CD206 (1:200, Proteintech, China) and anti-TNF-α (1:200, Proteintech, China) at 4 °C overnight, followed by Alexa Fluor 488 (1:1000, Abcam, UK) or Alexa Fluor 555 (1:1000, Abcam, UK) for 2 h at RT. The results were examined under a fluorescence microscope (OLYMPUS, USA).

## 3 Statistical analysis

All experiments were performed with 3 replicates and repeated at least 3 times. Data were presented as mean ± standard deviation (SD). Differences between control and experimental groups were analyzed using a one-way analysis of variance (ANOVA), followed by Tukey post hoc comparison (GraphPad Software 9.0, CA, USA; Origin Pro 9.0 software, MA, USA). A value of *p* < 0.05 denotes statistically significant difference.

## 4 Results

### 4.1 Preparation and characterization of hydrogels

**Fig. 1:**
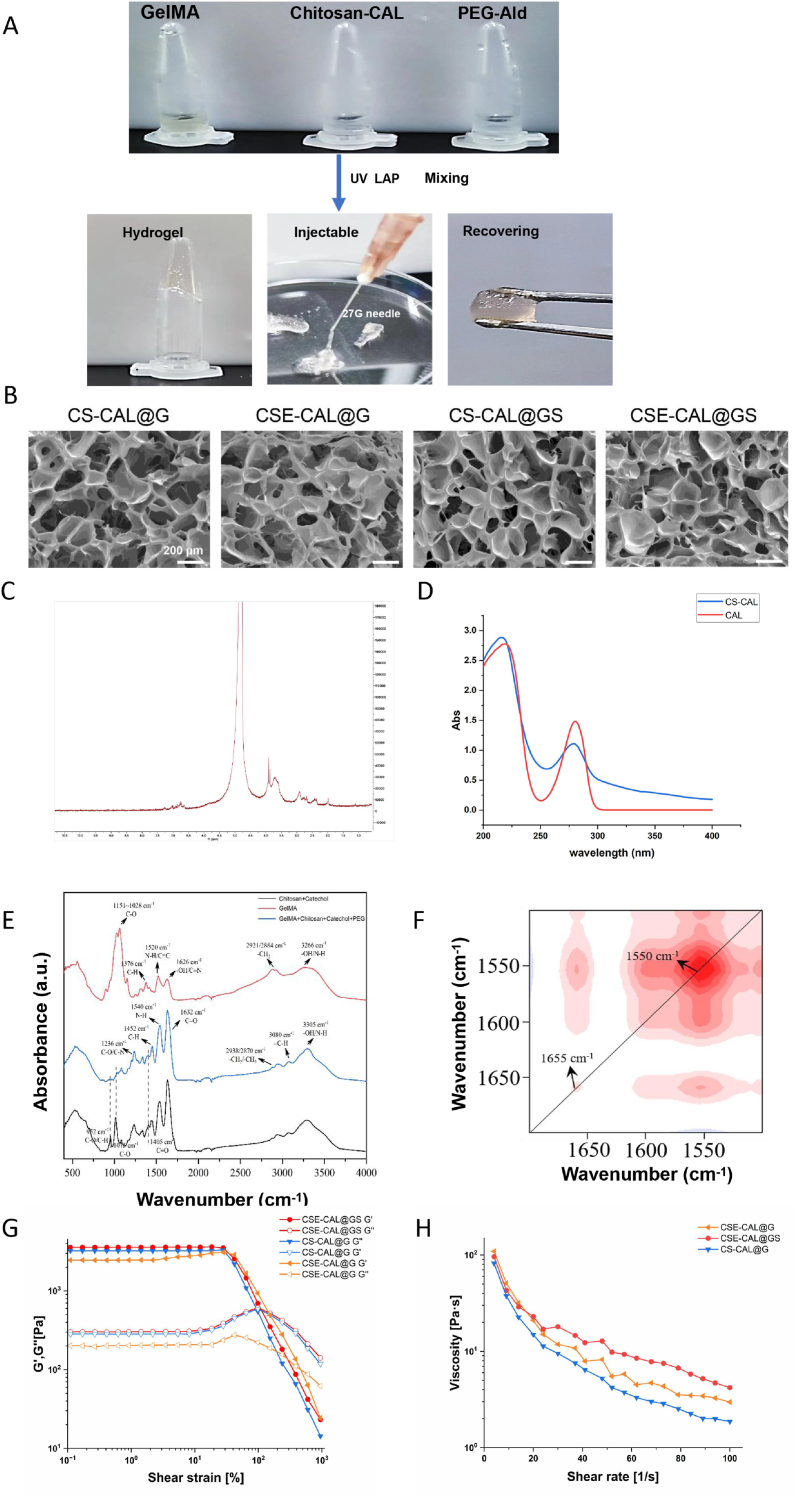
Synthesis and characterization of composite hydrogels. (A) Schematic illustration of the hydrogel synthesis process. (B) SEM images of composite hydrogels. (C) Use ^1^ H NMR spectra to test CS-CAL. (D) Use of the UV–vis test CS-CAL and CAL. (E) FTIR spectra. Black arrows indicate specific stretching vibrations and bending vibrations. (F) 2DCOS spectra of amide bands in GelMA samples and GelMA/chitosan/polyphenol/PEG composite samples. (G) Amplitude sweep of hydrogels with shear strain ranging from 0.1 % to 1000 %. (H) Viscosity of the hydrogels plotted against the shear rate. ∗∗∗*P* < 0.001.

The composite hydrogel system, consisting of GelMA/Chitosan-CAL/PEG-Ald, was first crosslinked through ultraviolet (UV) photopolymerization and then engineered for controlled delivery via syringe injection (Fig. 1A). Furthermore, SEM was used to analyze the microporous structure of hydrogels with different components. All hydrogel formulations exhibited comparable interconnected porous architectures, demonstrating that the incorporation of either CS or supplementary components exerted negligible influence on the structural integrity of the GelMA-based crosslinked network (Fig. 1B). Notably, the porous structure provides a more favorable microenvironment for cell adhesion, proliferation, and migration. To chemically characterize the hydrocaffeic acid-functionalized chitosan (CS-CAL), we performed ^1^H NMR and UV–Vis spectroscopy. In the ^1^H NMR spectrum, new signals corresponding to the aromatic protons of the phenolic moiety were observed in the range of 6.8–6.5 ppm (Fig. 1C). UV–Vis spectroscopy, with which we examined both pure hydrocaffeic acid and chitosan-hydrocaffeic acid, further corroborated this modification. The spectra of both samples were generally consistent, with the main difference being a slight elevation in the baseline for the chitosan-hydrocaffeic acid. The degree of substitution (DS) of caffeic acid groups was quantified as 60 % using a UV–Vis calibration curve with caffeic acid as the standard (Fig. 1D and S2). Collectively, these data confirm the successful synthesis of caffeic acid-grafted chitosan.

The chemical cross-linking of GelMA/Chitosan-CAL/PEG-Ald was verified using FTIR. The relative intensity of the absorption peak at 1015 cm^−1^ and 952 cm^−1^ increased significantly, indicating that chitosan and hydrocaffeic acid were successfully introduced into the GelMA. The synchronous spectra showed that the C=O groups underwent condensation reactions with N-H groups (Fig. 1E and F).

To further elucidate the viscoelastic behavior of the hydrogels, rheological analyses were conducted both before and after gelation. Frequency sweep measurements within the linear viscoelastic region (LVR) revealed the evolution of storage modulus (G′) and loss modulus (G″) as a function of frequency (Fig. S3A and S3B). Prior to gelation, the hydrogels exhibited relatively low moduli with weak frequency dependence, indicating a sol-like state. In contrast, after gelation, both G′ and G″ increased markedly and demonstrated a pronounced frequency dependence, suggesting the establishment of a robust crosslinked network. To further characterize the rheological behavior, both pre-gel and post-gel states of the hydrogels were examined. Before gelation, amplitude sweep and steady shear tests (Fig. S4A and S4B) revealed relatively low storage modulus (G′) and loss modulus (G″), together with a rapid decrease in viscosity upon increasing shear rate, consistent with a sol-like state of the precursor solutions. In contrast, after gelation, the hydrogels exhibited an extensive linear viscoelastic region with markedly enhanced G′ values and pronounced shear-thinning behavior (Fig. 1G and H), indicating the formation of robust and reversible crosslinking networks. As shown in Video S1, the hydrogel exhibited excellent injectability as it was extruded from a syringe and placed in a petri dish. The advancement of biomaterials possessing shear-thinning and self-healing properties is pivotal for injectable applications, as injectability is a critical requirement for numerous biomedical applications.

To substantiate the tissue adhesiveness, hydrogels with different compositions were subjected to tensile testing, displaying typical elastomeric stress–strain profiles with a distinct linear elastic region, yielding, and an elongation at break of ∼86 % under 100 N loading (Fig. S5A–5B). The relatively low modulus confirmed a compliant yet mechanically stable network, ensuring high stretchability and tensile integrity to support interfacial adhesion. Under physiological conditions, robust adhesion was further demonstrated by firm attachment of the hydrogel to a rat knee joint, which resisted mechanical lifting (Fig. S5C, Video S2–S3), while cut pieces readily self-healed within 25 min to form an integrated construct that maintained integrity under gravity (Fig. S5D). These findings highlight that the hydrogels integrate mechanical resilience with strong bioadhesion, thereby enabling secure immobilization on cartilage surfaces for effective tissue repair.

### 4.2 Potential of functionalized microspheres as a delivery platform

**Fig. 2:**
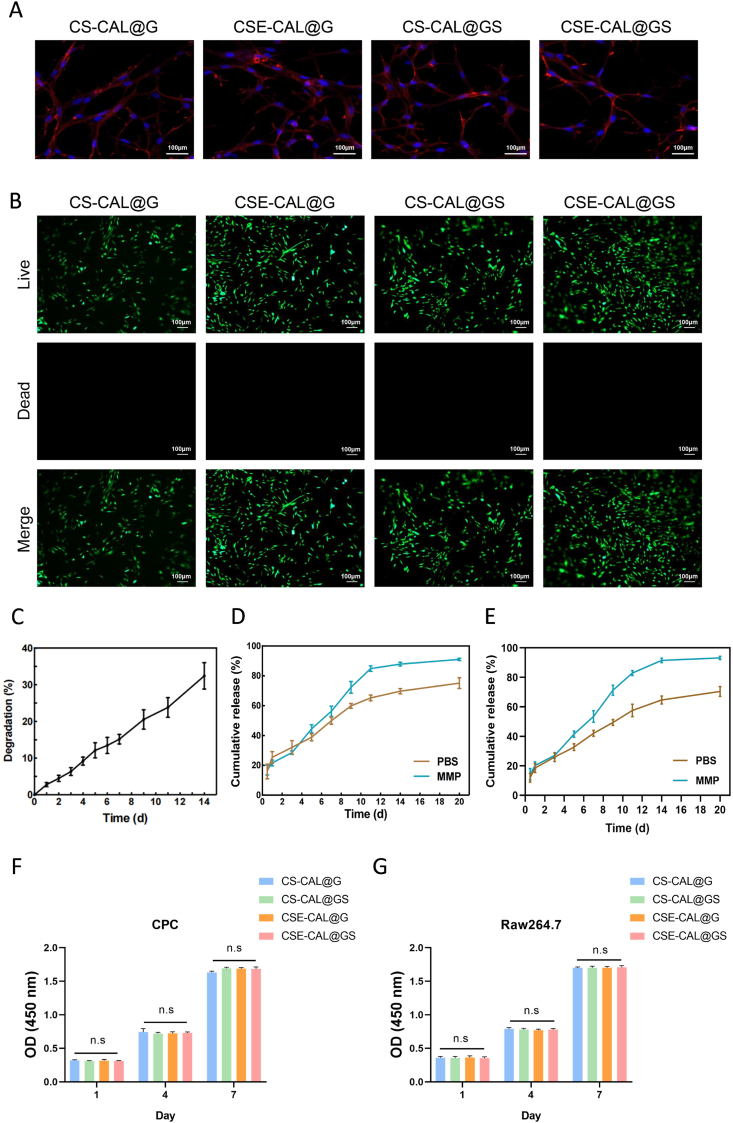
Biocompatibility and functional characterization of the composite hydrogel. (A) Cytoskeletal staining of CPCs. (B) Live/dead staining results of CPCs cultured in different hydrogels. (C) Degradation profile of the composite hydrogel. (D–E) Release profiles of SDF-1α and EGTA. (F–G) Cell viability of hydrogels in CPCs and RAW264.7 cells after gelation at different time points. N.S., not significant.

Biocompatibility is an essential property of implantable biomaterials in the body. Cytoskeletal staining showed that CPCs spread effectively on the hydrogel (Fig. 2A). Additionally, there were only a small number of dead cells in all four groups (Fig. 2B), which suggested that all these groups exhibited good cytocompatibility, and no significant differences among the groups. Furthermore, in simulated physiological conditions, rapid molecular diffusion through the hydrogel network and slow degradation were observed over 2 weeks. However, in the presence of MMPs, 98 % of the hydrogel was within 5 days (Fig. 2C and S6).

SDF-1α was evaluated in PBS and MMP, respectively. The results verified that the release kinetics of SDF-1α was related to MMP. By day 11, the release rates of SDF-1α were 88 % and 66 %, respectively (Fig. 2D). When incubated in calcium-containing solutions, the composite hydrogels demonstrated progressive calcium ion depletion, reflecting EGTA release kinetics. In response to the influence of MMP overexpressed in the arthritic microenvironment, the hydrogels exhibited a distinct biphasic release profile: an initial rapid phase (≤11 days) with accelerated calcium decline, followed by sustained slower release (Fig. 2E). The initial rapid release phase can be attributed to the hydrogel's responsiveness to MMP, which cleaves crosslinkers within the hydrogel matrix, thereby facilitating the release of both SDF-1α and EGTA. This demonstrates the MMP-responsive drug delivery properties of the GelMA hydrogel. The CCK-8 assay confirmed that both the pre-gel solutions and the crosslinked hydrogel groups exhibited good cytocompatibility. No significant differences in cell viability were observed among the pre-gel groups, and similarly, all hydrogel groups after gelation maintained high cell viability at all examined time points (Fig. 2F-G, and S7A-7B).

### 4.3 ROS-scavenging and anti-inflammation of CSE-CAL@GS in CPCs

In this study, synovial fluid samples were collected from the knee joints of patients with RA or OA. Afterwards, the calcium ion concentration of the samples was measured (Fig. S8). Our results showed that the calcium ion concentration was higher in synovial fluid samples from the knee joints of patients with RA than in samples from patients with OA. This pathological elevation of extracellular calcium in the arthritic joint microenvironment prompted us to develop a responsive hydrogel system capable of calcium sequestration and controlled drug release.

**Fig. 3:**
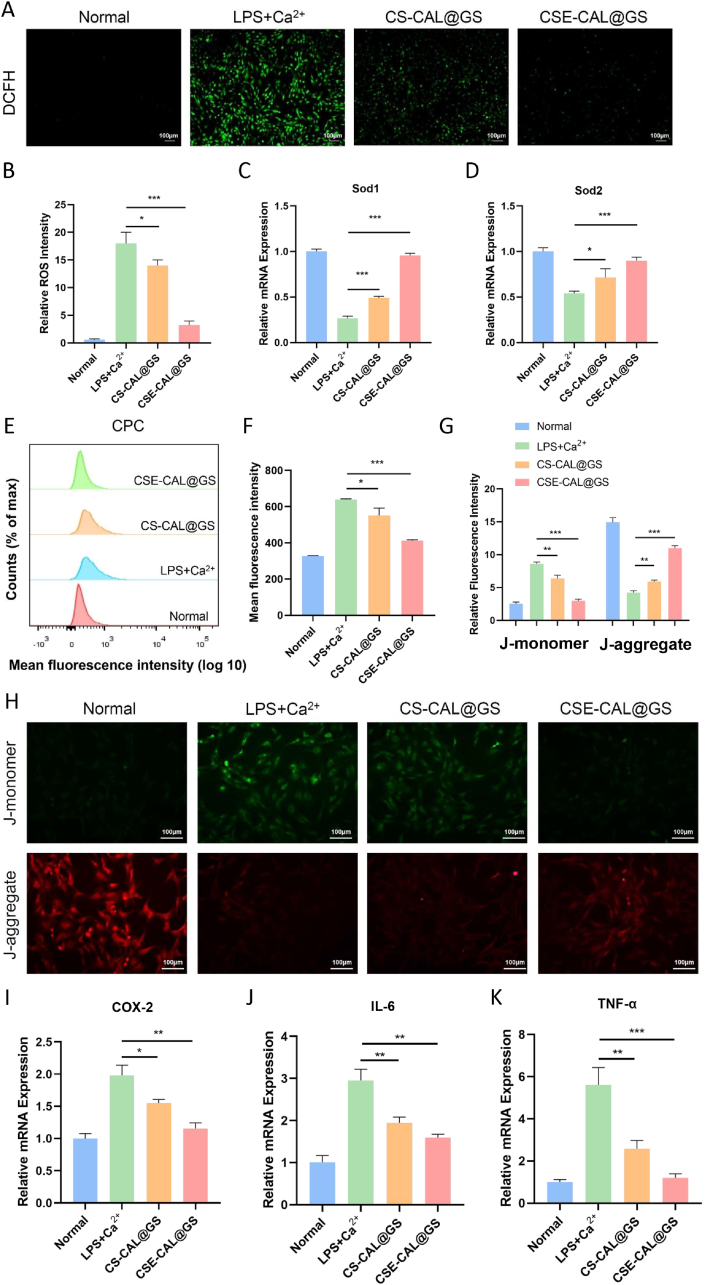
The effect of composite hydrogels on ROS scavenging in CPCs. (A–B) H2DCFH- DA, a common ROS probe, was used to evaluate the antioxidant capability of CS-CAL@GS and CSE-CAL@GS. (C–D) Expression of antioxidant enzyme genes in CPCs evaluated by qRT-PCR. (E–F) Intracellular ROS levels detected by H2DCFH-DA staining and measured via FCM. (G–H) Fluorescence microscope images of JC-1 assay demonstrating activated (labeled red) and abnormal (labeled green) mitochondrial membrane potential. (I–K) Effects of CSE-CAL@GS on IL-6, TNF-α, and COX-2 mRNA levels in chondrocytes as determined by qRT-PCR. N.S., not significant; ∗*P* < 0.05, ∗∗*P* < 0.01, and ∗∗∗*P* < 0.001. (For interpretation of the references to colour in this figure legend, the reader is referred to the Web version of this article.)

As shown in Fig. S9A–D, the chitosan-only group exhibited no significant anti-ROS activity, indicating its limited effectiveness in ROS scavenging. Therefore, this group was excluded from subsequent experimental groups to focus on more effective formulations. Following LPS and Ca^2+^ stimulation, CPCs exhibited significantly reduced intracellular ROS levels upon treatment with either CS-CAL@GS or CSE-CAL@GS hydrogels (Fig. 3A and B). Flow cytometry analysis supported these findings (Fig. 3E and F).

Compared to the control group, CS-CAL@GS and CSE-CAL@GS hydrogels significantly upregulated the expression of the antioxidant genes superoxide dismutase 1 (Sod1) and superoxide dismutase 2 (Sod2) (Fig. 3C and D). JC-1 staining, a sensitive fluorescent probe for monitoring mitochondrial membrane potential (ΔΨm), was employed to assess mitochondrial integrity, as its fluorescence shift from red (J-aggregates) to green (monomers) reflects early apoptotic changes. In an inflammatory milieu, CSE-CAL@GS treatment enhanced mitochondrial membrane potential as demonstrated by increased JC-1 red fluorescence intensity. This partial restoration suggests the therapeutic potential of CSE-CAL@GS for mitigating inflammation-induced mitochondrial dysfunction (Fig. 3G and H). Furthermore, qRT-PCR analysis demonstrated that CSE-CAL@GS significantly downregulated the expression of COX-2, IL-6, and TNF-α in cells stimulated with LPS and Ca^2+^ (Fig. 3I–K). These findings confirm the composite hydrogel's anti-inflammatory and antioxidant effects.

### 4.4 Intracellular radical scavenging capability of composite hydrogels in RAW264.7

**Fig. 4:**
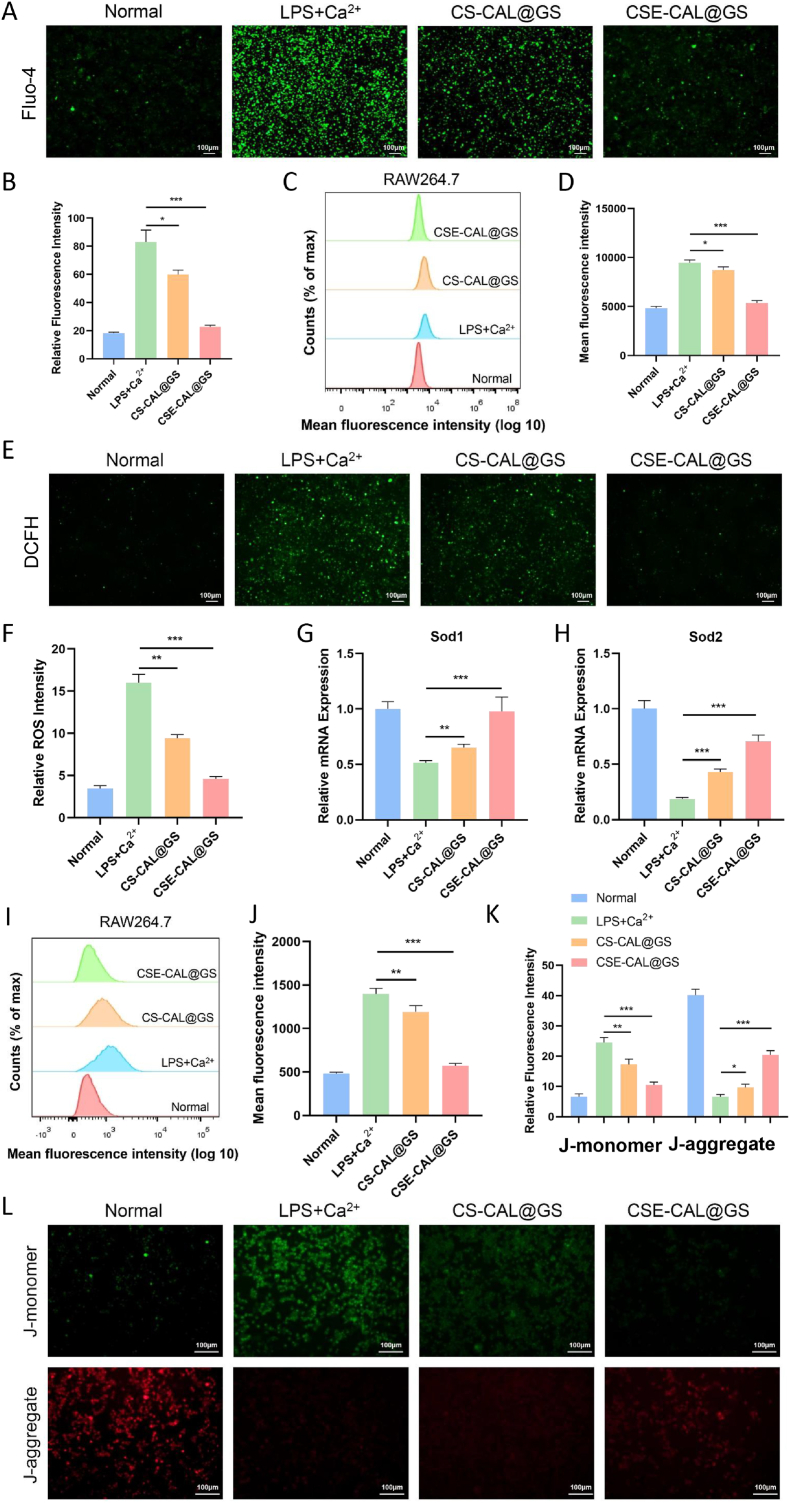
Effects of composite hydrogels on ROS levels and mitochondrial function in RAW264.7 cells. (A–B) Fluo-4 AM staining was used to visualize intracellular Ca^2+^ in LPS + Ca^2+^-induced M1 RAW264.7 macrophages. Untreated RAW264.7 cells served as the normal control. Semiquantitative fluorescence intensity analysis was performed using ImageJ. (C–D) The changes of Ca^2+^ concentration in M1 macrophages induced by LPS + Ca^2+^ were detected by flow cytometry. (E–F) H2DCFH-DA, a common ROS probe, was used to evaluate the ROS scavenging ability of CS-CAL@GS and CSE-CAL@GS. (G–H) Expression of antioxidant enzyme genes (Sod1 and Sod2) in RAW264.7 cells was evaluated by qRT-PCR. (I–J) Intracellular ROS levels were measured using H2DCFH-DA staining and flow cytometry. (K–L) Fluorescence microscope images of JC-1 assay demonstrating activated (labeled red) and abnormal (labeled green) mitochondrial membrane potential. ∗*P* < 0.05, ∗∗*P* < 0.01, and ∗∗∗*P* < 0.001. (For interpretation of the references to colour in this figure legend, the reader is referred to the Web version of this article.)

To assess inflammation-associated calcium dysregulation, we quantified intracellular Ca^2+^ dynamics in LPS-stimulated RAW264.7 cells. Quantitative analysis combining flow cytometry and live-cell fluorescence imaging demonstrated a marked elevation in intracellular Ca^2+^ levels following LPS stimulation. While the influx appeared to noticeably decrease after treatment with the composite hydrogel (Fig. 4A–D). Furthermore, we used H2DCFH-DA to assess ROS levels in LPS + Ca^2+^-treated RAW264.7 cells, and observed the weakest intracellular fluorescence intensity after CSE-CAL@GS treatment. These findings suggest that CSE-CAL@GS exhibits a more robust capacity for ROS scavenging than CS-CAL@GS (Fig. 4E and F).

Compared with the LPS + Ca^2+^ treatment group, the fluorescence peak of CSE-CAL@GS significantly shifted to the left, indicating a reduced number of ROS-positive cells in CSE-CAL@GS (Fig. 4I and J). qRT-PCR analysis revealed significantly decreased expression of Sod1 and Sod2 in LPS/Ca^2+^-treated RAW264.7 cells compared to all other experimental groups (Fig. 4G and H). This suppression of antioxidant enzymes correlates with elevated ROS levels, suggesting impaired oxidative stress defense mechanisms. CSE-CAL@GS treatment attenuated this effect, indicating its potential to reestablish redox homeostasis. Complementary assessment of mitochondrial membrane potential using JC-1 fluorescence further supported these findings. During inflammatory stimulation, both CS-CAL@GS and CSE-CAL@GS produced modest increase in mitochondrial membrane potential, consistent with partial restoration toward baseline (Fig. 4K and L). The composite hydrogel exhibited significant anti-inflammatory and antioxidant activity in RAW264.7 cells, as evidenced by suppression of pro-inflammatory cytokines and reduction of reactive oxygen species.

### 4.5 Effects of composite hydrogels on inflammation through immunomodulation

**Fig. 5:**
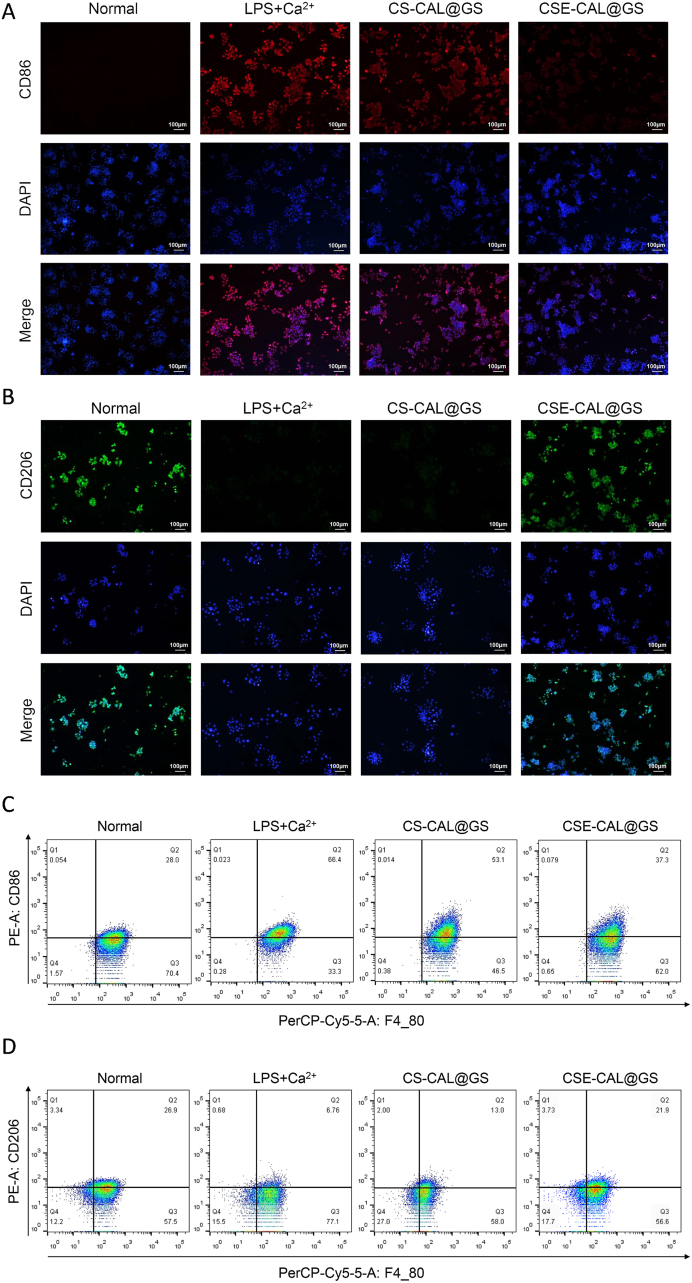
Identification of macrophage polarization by immunofluorescence and flow cytometry. (A) Immunofluorescence staining of CD86^+^ (M1) macrophages in RAW264.7 cells. (B) Immunofluorescence staining of CD206^+^ (M2) macrophages in RAW264.7 cells. (C–D) Flow cytometric analysis of CD86^+^ (M1) and CD206^+^ (M2) macrophages.

Moreover, we assessed its ability to reprogram M1 macrophages to an M2 phenotype. Macrophage polarization was assessed in CSE-CAL@GS-treated RAW264.7 cells using immunofluorescence staining of CD86 (M1 marker, red) and CD206 (M2 marker, green). After co-stimulation with LPS + Ca^2+^, macrophages demonstrated robust red fluorescence (Fig. 5A and S10A), confirming predominant M1 polarization. In contrast, the red fluorescence intensity of LPS + Ca^2+^-treated RAW264.7 cells cultured with CSE-CAL@GS significantly decreased, while the green fluorescence intensity increased significantly (Fig. 5B and S10B), indicating that CSE-CAL@GS repolarized a large proportion of M1 macrophages to the M2 phenotype.

Flow cytometric analysis demonstrated distinct effects of CSE-CAL@GS hydrogel on macrophage polarization in the LPS + Ca^2+^-induced inflammatory cellular model. Compared to LPS + Ca^2+^ treatment group, the CSE-CAL@GS group showed decreased M1 polarization (F4/80^+^CD86^+^), though this remained higher than untreated baseline levels (Fig. 5C and S10C). Conversely, CSE-CAL@GS treatment enhanced M2 polarization (F4/80^+^CD206^+^), showing increased polarization compared to LPS + Ca^2+^ treatment group (Fig. 5D and S10D). These findings suggest that hydrocaffeic acid and EGTA modifications contribute to the immunomodulatory capacity of the composite hydrogel by promoting M2 polarization. To further assess macrophage polarization following LPS + Ca^2+^ challenge, we quantified cytokine secretion by ELISA. TNF-α levels increased markedly in response to LPS + Ca^2+^, indicating enhanced M1 polarization. Treatment with CS-CAL@GS significantly reduced TNF-α secretion, whereas CSE-CAL@GS produced a further decrease, suggesting greater suppression of M1-associated cytokine production (Fig. S11A). Conversely, IL-10 (a marker of M2 polarization) was elevated in both CS-CAL@GS and CSE-CAL@GS groups compared with the LPS + Ca^2+^ group, with CSE-CAL@GS showing a more pronounced increase (Fig. S11B). These results support that both CS-CAL@GS and CSE-CAL@GS promote macrophage polarization toward an anti-inflammatory M2 phenotype, with CSE-CAL@GS exhibiting superior efficacy.

### 4.6 The protective effects of composite hydrogels on mitochondrial function and calcium homeostasis in aging cells

Mitochondria play a pivotal role in cellular aging, particularly in the imbalance between mitochondrial fission and the aging process. Excessive mitochondrial fission is often accompanied by mitochondrial fragmentation and functional deterioration, which are strongly associated with oxidative stress, disrupted energy metabolism, and calcium overload. Moreover, calcium overload is a prevalent phenomenon in aging cells. Excessive calcium influx into the mitochondria compromises the integrity of the inner mitochondrial membrane, resulting in swelling, functional decline, and enhanced oxidative stress, which collectively accelerate cellular aging [18].

**Fig. 6:**
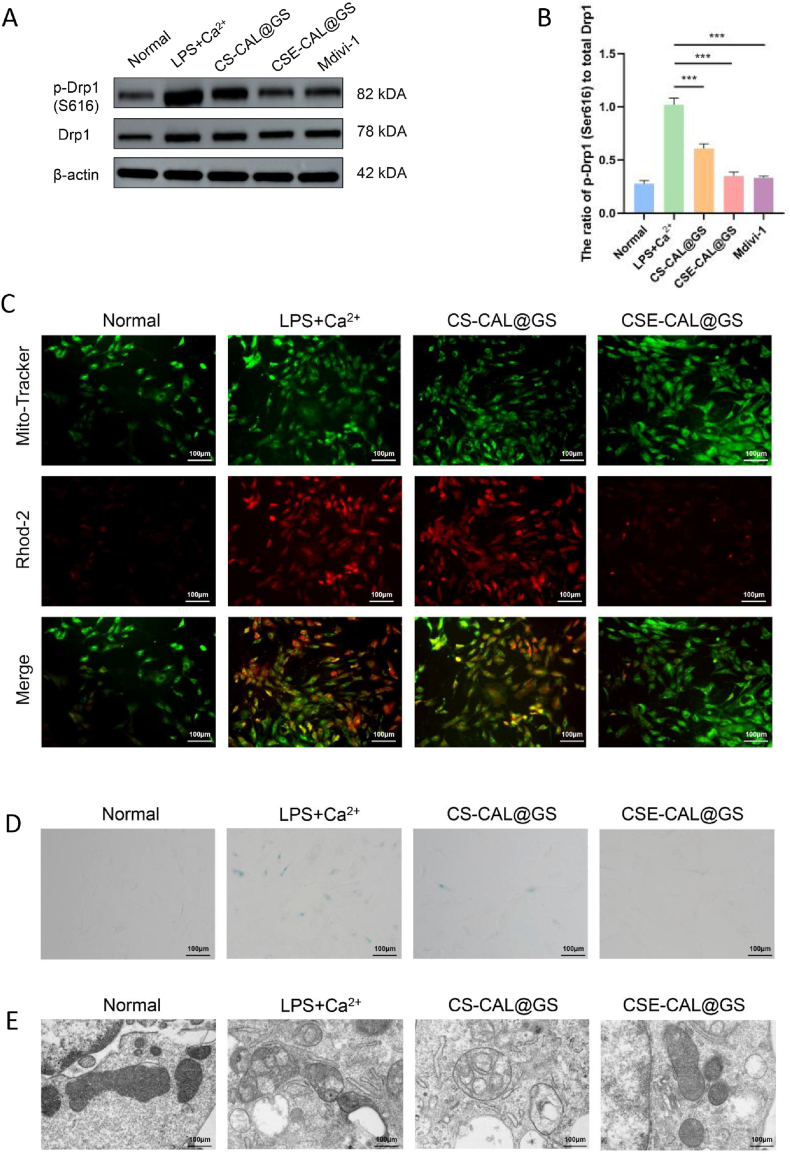
Composite hydrogel reduces mitochondrial calcium overload and improves mitochondrial function in CPCs. (A–B) Western blot analysis of p-Drp1 expression. (C) Representative fluorescent images of CPCs stained with MitoTracker Green (mitochondria, green) and Rhod-2 AM (mitochondrial calcium, red). (D) SA-β-gal staining of CPCs from the four experimental groups. (E) Morphology of mitochondria detected by transmission electron microscopy. ∗*P* < 0.05 and ∗∗∗*P* < 0.001. (For interpretation of the references to colour in this figure legend, the reader is referred to the Web version of this article.)

To systematically evaluate the therapeutic potential of the composite hydrogel against cellular senescence, we examined both total Drp1 and its phosphorylation at Ser616 by Western blot (Fig. 6A and B). The LPS + Ca^2+^ group exhibited a significant increase in the p-Drp1/Drp1 ratio compared with the Normal group, indicating enhanced Drp1 activation and mitochondrial fission. In contrast, treatment with CSE-CAL@GS markedly reduced this phosphorylation ratio, similar to the effect of Mdivi-1, a selective Drp1 inhibitor used as a positive control. These findings suggest that CSE-CAL@GS mitigates Drp1 activation by decreasing Ser616 phosphorylation, thereby alleviating excessive mitochondrial fission and cellular senescence.

Additionally, the expression of COX-4, a key subunit of mitochondrial respiratory complex IV, was markedly down-regulated, suggesting impaired oxidative phosphorylation (Fig. S12A–B). Given that mitochondrial calcium overload is a known trigger of bioenergetic dysfunction, we further assessed calcium homeostasis using western blot and Rhod-2-based fluorescence imaging. IP3R (inositol-1,4,5-trisphosphate receptors) protein levels were quantified by densitometry of Western blots, normalized to β-actin, across the experimental groups (Fig. S13A–B). IP3R protein expression peaked in the LPS + Ca^2+^ group, consistent with robust upregulation of the ER Ca^2+^-release channels when inflammatory signaling coincided with Ca^2+^ overload. In contrast, the CSE-CAL@GS treatment group showed a marked reduction in IP3R expression compared with the LPS + Ca^2+^ group, suggesting a protective effect by mitigating aberrant IP3R upregulation. In the CSE-CAL@GS group, IP3R expression was comparable to that in the Normal group, suggesting that the intervention effectively restored IP3R expression toward baseline levels. These results suggest that the therapeutic benefits of CSE-CAL@GS may , at least in part, stem from modulation of ER Ca^2+^ release, which in turn attenuates mitochondrial Ca^2+^ overload and related cellular injury. Consistent with this mechanism, mitochondrial Ca^2+^ accumulation, assessed by Rhod-2 fluorescence, was significantly elevated in the LPS + Ca^2+^ group, whereas CSE-CAL@GS treatment effectively attenuated this increase (Fig. 6C and S14). Thus, modulation of IP3R-mediated ER Ca^2+^ release translated into reduced mitochondrial Ca^2+^ overload, which in turn alleviated downstream bioenergetic impairment and cellular injury.

Notably, CSE-CAL@GS hydrogel treatment not only attenuated LPS/Ca^2+^-mediated Drp1 upregulation but also restored physiological mitochondrial calcium homeostasis, whereas CS-CAL@GS showed only partial efficacy in calcium modulation. These effects are likely mediated through hydrocaffeic acid-dependent suppression of inflammatory signaling pathways, thereby preventing aberrant mitochondrial calcium accumulation. Substantiating these findings, β-galactosidase staining confirmed that CSE-CAL@GS significantly reduced LPS/Ca^2+^-induced SA-β-gal activity in CPCs (Fig. 6D and S15).

Furthermore, transmission electron microscopy (TEM) was used to investigate the impact of the treatment on mitochondrial ultrastructure. In the normal group, mitochondria exhibited a well-preserved, filamentous morphology, whereas the LPS group showed prominent mitochondrial fragmentation and swelling. Importantly, these structural abnormalities were alleviated in the treatment groups, restoring a more uniform, filamentous mitochondrial network (Fig. 6E).

The composite hydrogel delays cellular aging by attenuating excessive mitochondrial fission through Drp1 downregulation, restoring calcium homeostasis to prevent membrane dysfunction, and preserving oxidative phosphorylation via COX-4 stabilization. Together with its established ROS-scavenging and M2 macrophage-polarizing properties, the hydrogel coordinately addresses key senescence drivers—oxidative stress, inflammatory signaling, and metabolic impairment—thereby providing a multifaceted defense against cellular aging.

### 4.7 Cell migration and chondrogenesis differentiation

**Fig. 7:**
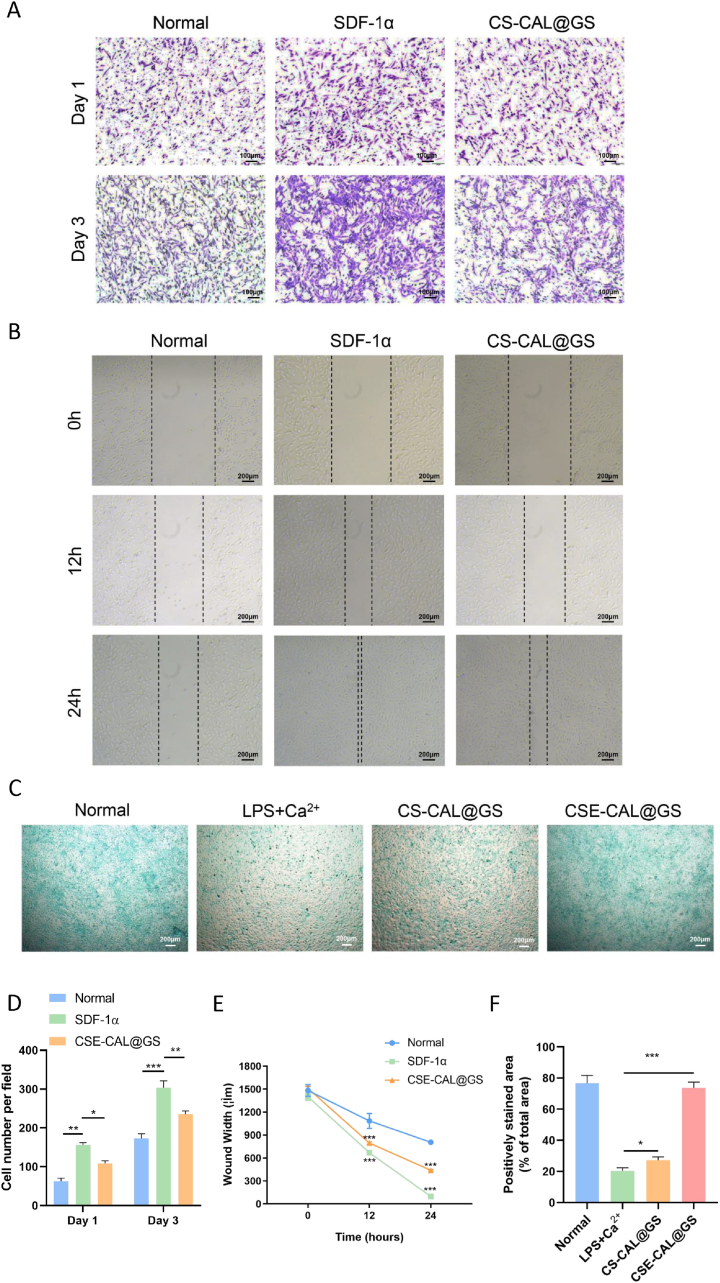
SDF-1α promotes CPCs migration and chondrogenic differentiation. (A and D) Representative images of CPCs migration in transwell assays and quantification of migrated cells following SDF-1α treatment. (B and E) Optical images of wound healing assays and their quantitative analysis. (C and F) Alcian Blue staining of CPCs after 7 days of chondrogenic induction and semi-quantitative analysis of positively stained areas. ∗*P* < 0.05 and ∗∗∗*P* < 0.001. (For interpretation of the references to colour in this figure legend, the reader is referred to the Web version of this article.)

Cell migration assays revealed a significantly higher number of migrated cells in both the SDF-1α and CSE-CAL@GS groups compared to the normal controls after 24 h and 72 h (Fig. 7A–D). Notably, while the SDF-1α group exhibited a larger migration area than the CSE-CAL@GS group, both treatments demonstrated robust chemotactic effects. Consistent with these observations, scratch assays further confirmed that SDF-1α effectively enhances cell recruitment, while the hydrogel maintains excellent sustained-release properties (Fig. 7B–E). After 7 days in culture, Alcian Blue staining revealed markedly diminished proteoglycan deposition in CPCs co-treated with LPS and Ca^2+^ relative to normal controls. Treatment with CSE-CAL@GS partially restored cartilage matrix production in this inflammatory context (Fig. 7C–F). These findings highlight the therapeutic relevance of SDF-1α-loaded hydrogels for targeted cell recruitment and sustained delivery in cartilage regeneration strategies for RA.

### 4.8 Potential signaling pathway for the therapeutic effects of functionalized hydrogels

**Fig. 8:**
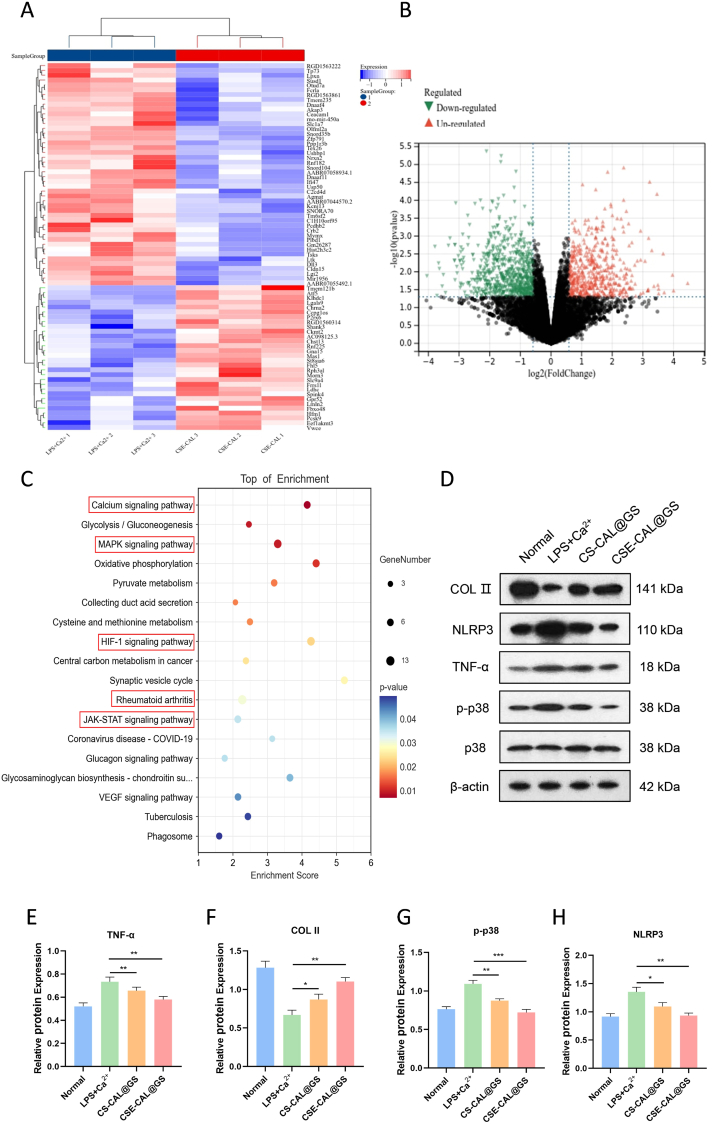
Transcriptomic analysis. (A) Heatmap and (B) Volcano plot showing differentially expressed genes (DEGs) between the CSE-CAL@GS group and the LPS + Ca^2+^ group. (C) KEGG enrichment bubble plot illustrating the top 20 pathways associated with the identified DEGs. (D–H) Western blotting of COL II, NLRP3, TNF-α, and p-p38 was performed, and semiquantitative analysis of band intensity was conducted using ImageJ software. ∗*P* < 0.05, ∗∗*P* < 0.01, and ∗∗∗*P* < 0.001.

To elucidate the molecular mechanisms underlying the therapeutic effects of the functionalized hydrogel, RNA-seq was performed on CPCs from the normal group and the CSE-CAL@GS group after 3 days of LPS + Ca^2+^ treatment. Heatmaps and volcano plots revealed differentially expressed genes (|log2FC|≥1 & p<0.05) in the CSE-CAL@GS group compared to the LPS + Ca^2+^ group (Fig. 8A and B). Furthermore, Gene Ontology (GO) functional enrichment analysis revealed potential target genes that were involved in the negative regulation of Ca^2+^ signaling and inflammatory responses (Fig. 8C).

Compared to the control group, the CSE-CAL@GS treatment group demonstrated significant downregulation of key signaling pathways, including MAPK and HIF-1α. Consistent with the RNA-seq findings, Western blot analysis further confirmed reduced expression of inflammatory mediators, such as P38 and TNF-α. Following LPS and Ca^2+^ stimulation, CPCs exhibited significant upregulation of p-p38 MAPK, TNF-α, and NLRP3 inflammasome components, which were substantially attenuated by CSE-CAL@GS treatment (Fig. 8D–H). Collectively, these findings suggest that the CSE-CAL@GS hydrogel exerts its therapeutic effects by suppressing LPS + Ca^2+^-induced hyperactivation of the MAPK/HIF-1α pathways, mitigating inflammatory responses (e.g., p-p38, TNF-α, and NLRP3 inflammasome), and restoring calcium homeostasis in CPCs, thereby offering a promising strategy for inflammatory-related mitochondrial dysfunction.

### 4.9 Therapeutic effect of the composite hydrogels on CIA rat

**Fig. 9:**
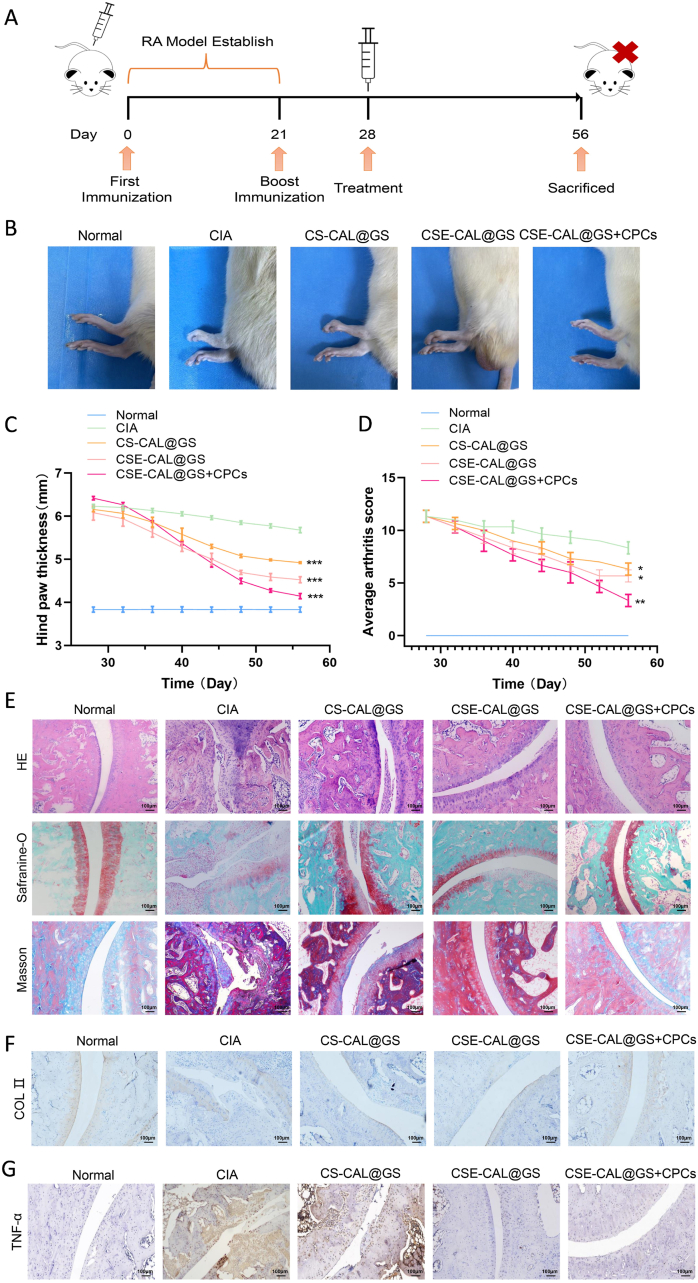
Histological evaluation of *in vivo* therapeutic outcomes. (A) The overall timeline for immunization and RA treatment. (B) Representative images of inflamed joints before and after different treatments. (C–D) Average arthritis scores for different groups throughout and at the end of the treatment. (E) Histological analysis of joint tissues using H&E, Masson, and Safranin O staining after various treatments. (F) Immunohistochemical staining of COL Ⅱ. (G) Immunohistochemical staining of TNF-α. ∗*P* < 0.05, ∗∗*P* < 0.01, and ∗∗∗*P* < 0.001.

To evaluate the therapeutic effect of CSE-CAL@GS + CPCs *in vivo*, we established a CIA rat model with similar pathological manifestations to human RA. CIA rat model was chosen because it recapitulates core RA immunopathology—loss of tolerance to type II collagen with synovitis, pannus formation, cartilage/bone erosion, and pro-inflammatory cytokine elevation—and shows pharmacologic responsiveness, providing a validated platform to assess intra-articular hydrogels and CPC-based therapy. This was achieved by administering subcutaneous injection of a mixture of bovine type II collagen and Freund's adjuvant into the tail of the rats according to previously published protocols. Clinical symptoms resembling RA were observed 28 days after the first immunization, confirming successful model establishment (Fig. 9A). *In vivo* retention of the composite hydrogel was assessed using the IVIS Spectrum imaging system. As shown in Fig. S16, the CSE-CAL@GS hydrogel group exhibited sustained bioluminescent signals within the joint cavity for up to 4 weeks post-injection.

To verify the therapeutic effect of chitosan-loaded hydrocaffeic acid, CIA rats were randomly divided into three groups and treated with CS-CAL@GS, CSE-CAL@GS and CSE-CAL@GS + CPCs, respectively. Among the treatment groups, rats receiving CSE-CAL@GS + CPCs exhibited minimal paw swelling and redness, closely resembling normal controls. The clinical arthritis score in the CSE-CAL@GS + CPCs treatment group was significantly lower than in the other groups, indicating superior therapeutic efficacy (Fig. 9B–D).

Furthermore, 56 days after immunization, the ankle joints of the rats were collected for histological analysis. H&E, Masson, and Safranin O staining showed that the model group had severe synovial hyperplasia, loss of glycosaminoglycans (GAGs), and cartilage damage (Fig. 9E). On the other hand, the CS-CAL@GS, CSE-CAL@GS, and CSE-CAL@GS + CPCs treatment groups showed improved histological characteristics. Among these, the CSE-CAL@GS + CPCs treatment group demonstrated the most pronounced therapeutic benefit, suggesting a synergistic effect between the hydrogel and stem cells. Sections of ankle joints stained with safranin O showed that the CSE-CAL@GS + CPCs group had more GAG deposition than other groups and the cartilage border was clearer and thicker, this was similar to the findings in the normal group. This indicates that the CSE-CAL@GS + CPCs treatment is an effective method for protecting cartilage when treating RA.

As shown in Fig. 9F and G, the expression of COL II in the CSE-CAL@GS hydrogel group was significantly higher than in both the CIA model group and CS-CAL@GS hydrogel groups. Additionally, TNF-α expression was significantly lower in the hydrogel-treated group than in the CIA model group. Given that SDF-1α can recruit CPCs and thereby further promote cartilage defect repair, the CSE-CAL@GS + CPCs hydrogel group exhibited the highest expression of COL II, indicating a favorable outcome in terms of cartilage regeneration.

**Fig. 10:**
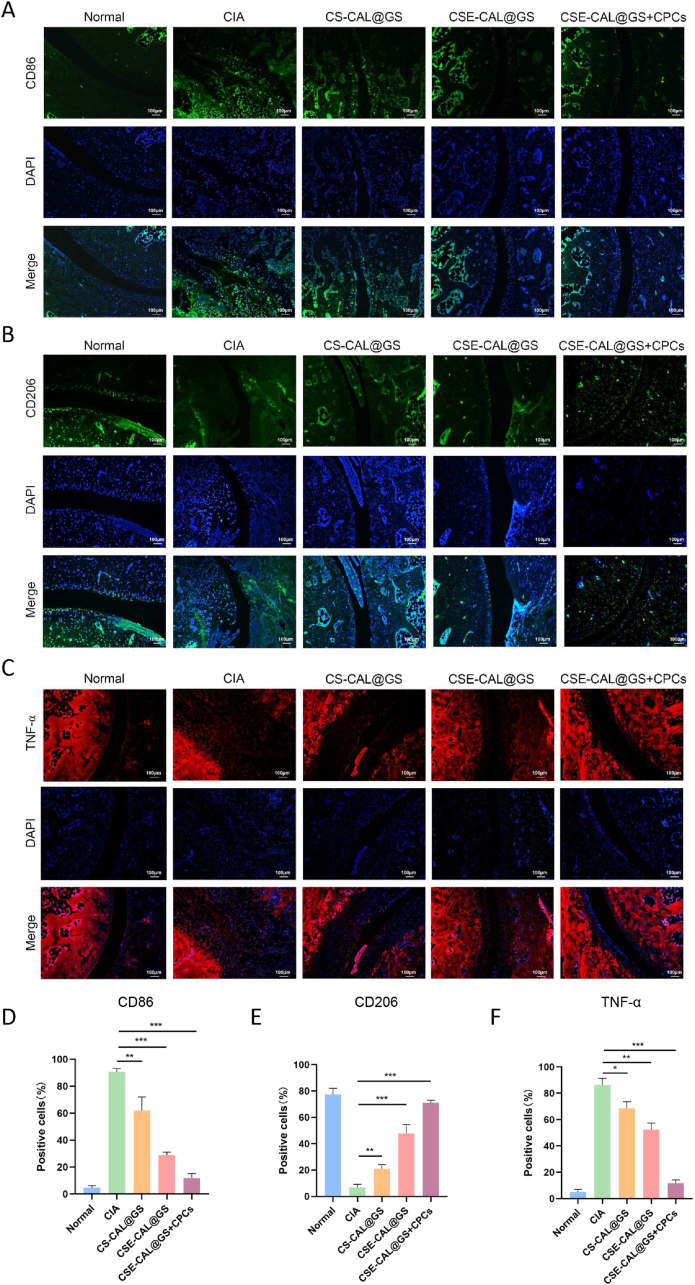
*In vivo* anti-inflammatory effects of the hydrogels. (A–C) Immunofluorescence staining of TNF-α, CD86, and CD206 in normal and inflamed joints following different treatments. (D–F) Semiquantitative analysis of fluorescence intensity was performed by ImageJ software. ∗∗*P* < 0.01, and ∗∗∗*P* < 0.001.

To determine the M1 to M2 phenotypic transition *in vivo*, we performed immunofluorescence staining for macrophage-specific biomarkers in the joints. Immunofluorescence staining showed that, compared with other treatments, CSE-CAL@GS + CPCs reduced CD86 (M1 biomarker) expression to near-normal levels, while upregulating CD206 (M2 biomarker) expression (Fig. 10A and B and 10D, E). TNF-α immunofluorescence staining further confirmed that CSE-CAL@GS + CPCs hydrogel treatment reduced local inflammation and facilitated *in vivo* M1 to M2 macrophage polarization (Fig. 10C–F).

**Fig. 11:**
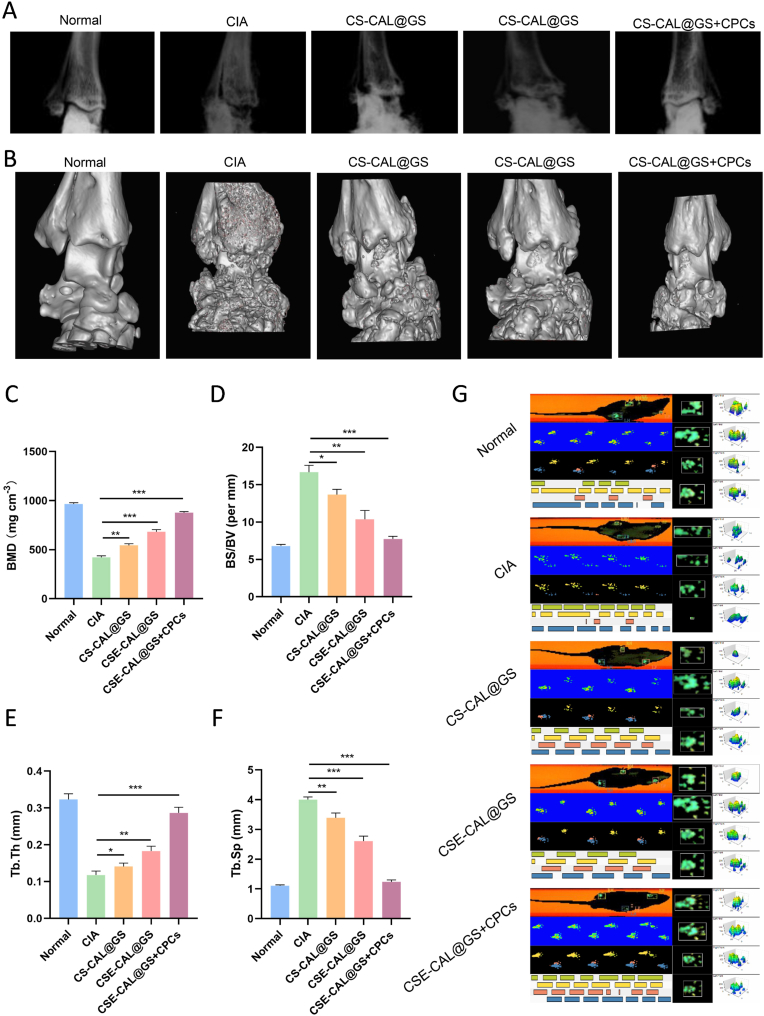
Evaluation of therapeutic efficacy following hydrogel treatment. (A) Representative X-ray images of joints from different groups. (B) Representative Micro-CT images of joints at day 28 following different treatments. (C) Bone mineral density (BMD), (D) bone surface to volume ratio (BS/BV), (E) trabecular thickness (Tb.Th) and (F) trabecular spacing (Tb.Sp) in different groups. (G) Representative footprints for analyzing the recovery of the limb motor function. (H–J) Quantitative comparison of maximum contact area, walking speed, and stride frequency across experimental groups. ∗*P* < 0.05, ∗∗*P* < 0.01, and ∗∗∗*P* < 0.001.

Bone erosion is a key pathological feature of RA. In this study, bone changes in the rats in different groups were assessed using plain radiographs and micro-computed tomography (Micro-CT). In the model group, inflamed ankle joints exhibited a rough and severely eroded bone surface (Fig. 11A and B), accompanied by markedly reduced bone mineral density (BMD) and trabecular thickness (Tb.Th) (Fig. 11C–E); however, they had increased bone surface area and trabecular spacing (Tb.Sp) compared to the control group (Fig. 11D–F). Locomotor functional recovery was quantitatively assessed through gait analysis. Compared to normal controls, CIA rats exhibited significant motor dysfunction, as evidenced by decreased walking speed, reduced maximum contact area and altered stride frequency, suggesting pain-related mobility impairment. Treatment with the composite hydrogel (CSE-GS@GS + CPCs group) demonstrated superior therapeutic efficacy, with walking speed nearly restored to normal levels (Fig. 11G). These findings highlight the remarkable potential of the composite hydrogel in alleviating arthritis-induced gait abnormalities.

## 5 Discussion

This study explores the potential of CSE-CAL@GS *in situ* gelation at the ankle joint as an immediate intervention in a CIA rat model. RA is characterized by synovial hyperplasia, cartilage degradation, and joint destruction, with chronic inflammation intensifying oxidative stress and metabolic imbalance [26]. This ultimately leads to mitochondrial dysfunction, including impaired oxidative phosphorylation, disrupted calcium homeostasis, and increased ROS production [27]. These abnormalities not only enhance joint inflammation but also hinder cartilage repair. At present, there is a lack of efficient therapeutic strategies to regulate mitochondrial function and restore the joint microenvironment. Biomaterial-based delivery systems have emerged as a hopeful approach, offering microenvironment regulation, mitochondrial function restoration, inflammation reduction, and cartilage regeneration. Consequently, the creation of innovative delivery platforms to strengthen mitochondrial function has become a key research focus in RA therapy.

In agreement with prior research, our study highlights the critical role of mitochondrial dysfunction, particularly calcium overload, in RA's pathogenesis. Brookes et al. have shown that abnormal accumulation of Ca^2+^ disrupts mitochondrial homeostasis. This disruption leads to abnormal opening of the mitochondrial permeability transition pore (mPTP) [28], triggering the release of cytochrome *c*, which subsequently causes excessive ROS accumulation due to the presence of a Ca^2+^ overload, a finding that aligns with our research (Fig. 3, Fig. 4E, F, I, J). In our study, we utilized EGTA incorporated into a CS hydrogel through electrostatic adsorption. EGTA reduces the influx of extracellular Ca^2+^ and curtails the excessive activation of the calcium-sensing receptor, effectively preventing mitochondrial Ca^2+^ overload (Fig. 4A and B), an observation in harmony with the findings of Jäger et al. [29]. Furthermore, EGTA restores intracellular calcium homeostasis, significantly reduces ROS levels, and inhibits the release of pro-inflammatory cytokines (Fig. 3I and J). These effects not only alleviate oxidative stress but also foster the transformation of pro-inflammatory M1 macrophages into anti-inflammatory M2 macrophages (Fig. 5), thereby easing RA-associated inflammatory responses.

Hydrocaffeic acid, a powerful antioxidant, effectively eliminates reactive ROS, reduces oxidative stress, and inhibits the expression of inflammatory mediators [30,31]. In this study, we successfully conjugated hydrocaffeic acid to the primary amine groups of CS using DMTMM, thus preserving their antioxidant properties and effectively maintaining mitochondrial homeostasis. Our findings reveal that the coordinated action of EGTA and hydrocaffeic acid significantly improved mitochondrial function, as demonstrated by attenuated calcium influx, reduced reactive oxygen species generation, and decreased inflammatory cytokine secretion (Fig. 3, Fig. 4). These results underscore the therapeutic potential of hydrocaffeic acid in treating RA, especially when considered for their synergistic effect with calcium homeostasis regulation, providing an efficient strategy for modulating the microenvironment of RA.

While EGTA and hydrocaffeic acid effectively regulate calcium homeostasis and oxidative stress, thus enhancing mitochondrial function, the dynamics of the mitochondria remain a critical factor in the pathogenesis of RA. Drp1, a member of the dynamin family of GTPase proteins, plays an essential role in mitochondrial fission [32]. Upon activation, Drp1 translocates to the mitochondria and initiates mitochondrial fragmentation [33]. Research conducted by You and others has demonstrated that the activation of Drp1 induces mitochondrial fission, which subsequently contributes to vascular cell senescence and the progression of atherosclerosis [34]. This finding is in line our study where it indicated that abnormal activation of Drp1 in an inflammatory environment disrupts mitochondrial homeostasis, resulting in the accumulation of senescent cells. Furthermore, our study revealed that a composite hydrogel containing EGTA and hydrocaffeic acid effectively mitigated the Drp1-induced mitochondrial fragmentation and the decline in mitochondrial membrane potential, thereby inhibiting cellular senescence (Fig. 7).

Furthermore, our hydrogel system incorporates SDF-1α to enhance the recruitment and retention of CPCs at sites of joint damage. SDF-1α is a chemokine recognized for its ability to recruit bone marrow mesenchymal stem cells (BMSCs) to areas of injury. Consistent with previous studies, SDF-1α not only facilitates the recruitment of BMSCs but also promotes the migration of CPCs [35,36]. Although therapies based on CPCs are promising for cartilage repair, their efficiency is often compromised by mitochondrial dysfunction within the RA microenvironment. Mitochondria play a vital role in the cartilage repair process. Zhu et al. have demonstrated that impairment of the mitochondrial respiratory chain function results in cartilage degradation and accelerates the progression of osteoarthritis [37]. By improving mitochondrial function, our hydrogels support chondrocyte viability and enhance cartilage repair (Fig. 6). This aligns with evidence suggesting that mitochondrial-targeted therapies can improve cartilage integrity in degenerative diseases, thereby strengthening the rationale behind our hydrogel design.

To further investigate the mechanisms underlying hydrogel-mediated mitochondrial protection, we carried out gene expression analysis and KEGG pathway enrichment. This revealed that calcium signaling, MAPK, JAK-STAT, and HIF-1 pathways are closely associated with inflammatory and oxidative stress responses in RA. Among these, p38MAPK signaling plays a pivotal role in regulating mitochondrial function and inflammation, as its excessive activation disrupts mitochondrial membrane potential, diminishes ATP synthesis, and increases oxidative stress [38]. Our results showed that LPS stimulation significantly increased p38 expression and phosphorylation in CPCs. However, functionalized hydrogel treatment successfully suppressed this activation. By inhibiting p38MAPK signaling, the hydrogel preserved mitochondrial integrity, decreased ROS production, and alleviated cartilage matrix degradation (Fig. 8). Previous studies also indicate that p38MAPK inhibition reduces inflammation and prevents joint degradation in RA models, further validating our findings [39]. Given its central role in oxidative stress regulation and mitochondrial dysfunction, targeting p38MAPK may be a promising therapeutic target for lessening inflammatory damage in RA. As Ca^2+^/ROS homeostasis and p38MAPK signaling govern T-cell activation and fate, the observed changes are likely to shape adaptive immunity [38,40,41]. Importantly, adaptive immunity—particularly CD4^+^ T cells—also shapes the synovial microenvironment and cartilage repair [42].

Specifically, Th1/Th17-skewed responses can sustain M1 polarization, amplify inflammatory and proteolytic cascades, and exacerbate chondrocyte dysfunction [43], whereas regulatory T cells (Tregs) favor resolution and matrix preservation [44]. Although CD4^+^ T cells were not profiled in this study, the observed normalization of Ca^2+^/ROS and suppression of p38MAPK suggest conditions that could influence CD4^+^ T-cell activation and subset balance. Clarifying these interactions lies beyond the scope of the present work and will be addressed in follow-up studies.

Histological analysis further corroborates the efficacy of our hydrogel, demonstrating increased cartilage tissue, better structural integrity, and improved collagen type II expression 8 weeks post-treatment (Fig. 9, Fig. 11). The observed repolarization from M1 to M2 macrophages contributes to the resolution of inflammation and tissue remodeling (Fig. 10). This is consistent with reports that M2 macrophages foster cartilage repair through the secretion of anti-inflammatory cytokines [45]. Imaging results and gait studies have exhibited the protective effects of the composite hydrogel on joint integrity. These findings underscore the potential of our hydrogel in reducing RA progression and enhancing joint regeneration. However, it is essential to conduct further investigations to confirm its long-term effects and optimize its use in clinical settings.

In conclusion, we engineered a nanocomposite hydrogel that combines covalent and non-covalent interactions, displays MMP sensitivity, and possesses calcium ion receptor-targeting capability for EGTA delivery. The micelles designed with hydrophobic cores effectively encapsulated SDF-1α. Together with SDF-1α, GelMA encapsulation resulted in a substantial increase of accumulation on the articular surface and a strong interaction with CPCs. However, there are some limitations to be acknowledged. First, while our study showcases promising *in vitro* and *in vivo* results, there's a need for long-term trials to assess the durability and stability of the hydrogel's effects. Another limitation is the lack of direct profiling of CD4^+^ T cells, which we will address by evaluating CD4^+^ T-cell composition and activation states in future work to refine the mechanistic model. Additionally, further investigation is required to fully understand the molecular mechanisms with which the hydrogel regulates mitochondrial function. Future research should emphasize optimizing the composition of the hydrogel and investigating its clinical translation potential. By integrating EGTA-mediated calcium, hydrocaffeic acid-mediated ROS scavenging, and p38MAPK inhibition, we introduced a comprehensive strategy to address RA pathology, particularly highlighting mitochondrial function and immune modulation. These findings corroborate the growing body of evidence suggesting that therapies targeting mitochondria present a promising strategy for RA treatment and cartilage regeneration.

## CRediT authorship contribution statement

**Jianxin Li:** Writing – review & editing, Writing – original draft, Methodology, Investigation, Data curation, Conceptualization. **Yingchen Ni:** Validation, Software, Methodology. **Yanjie Tao:** Validation, Formal analysis, Conceptualization. **Hao Cai:** Validation, Investigation, Conceptualization. **Anqi Wu:** Visualization, Validation. **Fengyuan Zhang:** Validation, Investigation. **Shijie Meng:** Visualization, Validation. **Yuyang Luo:** Validation, Software. **Weidong Zhang:** Writing – review & editing, Investigation, Funding acquisition, Formal analysis. **Youhua Wang:** Writing – review & editing, Visualization, Supervision, Project administration, Funding acquisition, Conceptualization.

## Funding

This study was supported by grants from Jiangsu Provincial Research Hospital (YJXYY202204), The Natural Science Foundation of 10.13039/501100002949Jiangsu Province (Grants No BK20241842), The National 10.13039/501100001809Natural Science Foundation of China (32401139).

## Declaration of competing interest

The authors declare that they have no known competing financial interests or personal relationships that could have appeared to influence the work reported in this paper.

## Data Availability

Data will be made available on request.
