# Supplementary material for: Functionalized hydrocaffeic acid-chitosan/EGTA hydrogel rescues mitochondrial dysfunction for immunomodulation and joint repair in rheumatoid arthritis
Source: Mater Today Bio. 2025 Nov 28;35:102605. doi: 10.1016/j.mtbio.2025.102605 (PMC12879476; doi:10.1016/j.mtbio.2025.102605)
Supplement: Multimedia component 1 [file mmc1.docx]

**Supplementary information**

| **Group Abbreviation** | **Full Designation & Composition** |
| --- | --- |
| ****CS-CAL@G**** | ****Chitosan-****hydrocaffeic acid****@GelMA**** • CS: Pure chitosan •CAL:hydrocaffeic acid-modified chitosan • G: Virgin GelMA (without SDF-1α) |
| ****CSE-CAL@G**** | ****Chitosan/EGTA-****hydrocaffeic acid****@GelMA**** • CSE: Chitosan + EGTA complex • CAL: hydrocaffeic acid-modified chitosan • G: Virgin GelMA |
| ****CS-CAL@GS**** | ****Chitosan-****hydrocaffeic acid****@GelMA/SDF-1α**** • CS: Pure chitosan • CAL: hydrocaffeic acid-modified chitosan • GS: GelMA + SDF-1α@PEG conjugate |
| ****CSE-CAL@GS**** | ****Chitosan/EGTA-****hydrocaffeic acid****@GelMA/SDF-1α**** • CSE: Chitosan/EGTA composite • CAL:hydrocaffeic acid-modified chitosan • GS: GelMA-SDF-1α delivery system |

**Figure S1**

**Figure S1**. Abbreviations and full names of key components in the hydrogel system.

**Figure S2**

**
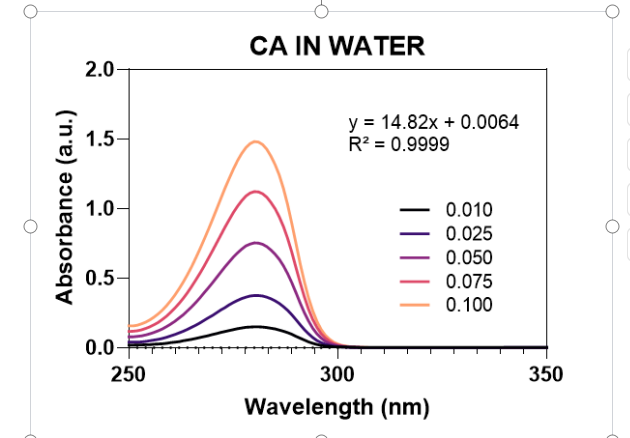
**

**Figure S2**. UV-vis spectra of CA in water at different concentrations (0.010–0.100 mg/mL). The calibration curve (y = 14.82x + 0.0064, R² = 0.9999) was used to calculate the grafting ratio.

**Figure S3**

**
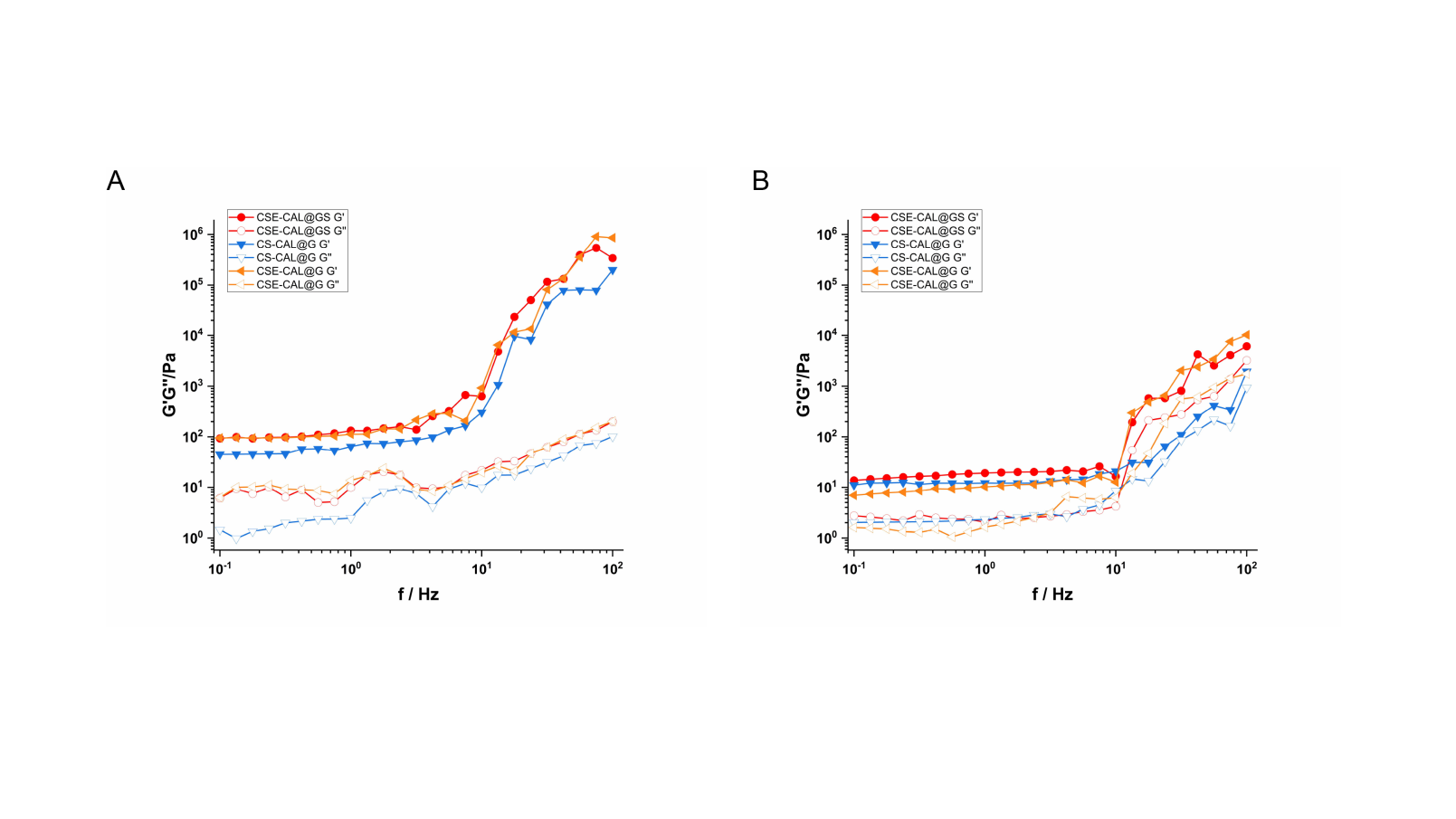
**

**Figure S3**. Frequency sweep measurements of hydrogels before (A) and after (B) gelation, showing storage modulus (G′) and loss modulus (G″) over 0.1–100 Hz in the linear viscoelastic region.

**Figure S4**

**
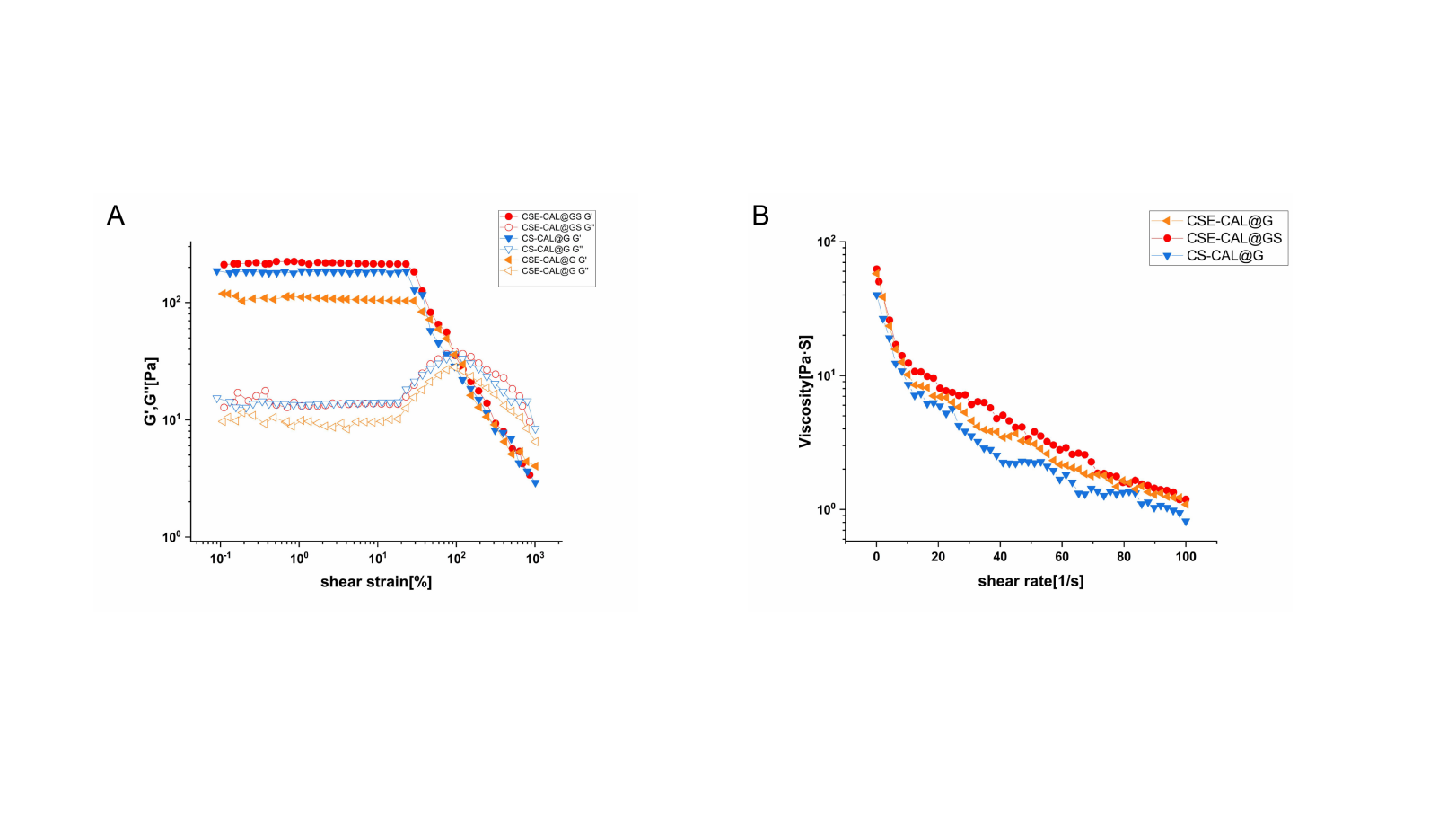
**

**Figure S4**. (A) Amplitude sweep of precursor hydrogels before gelation, showing storage modulus (G′) and loss modulus (G″) over shear strains ranging from 0.1% to 1000%. (B) Viscosity of the precursor hydrogels before gelation plotted against shear rate.

**Figure S5**

**
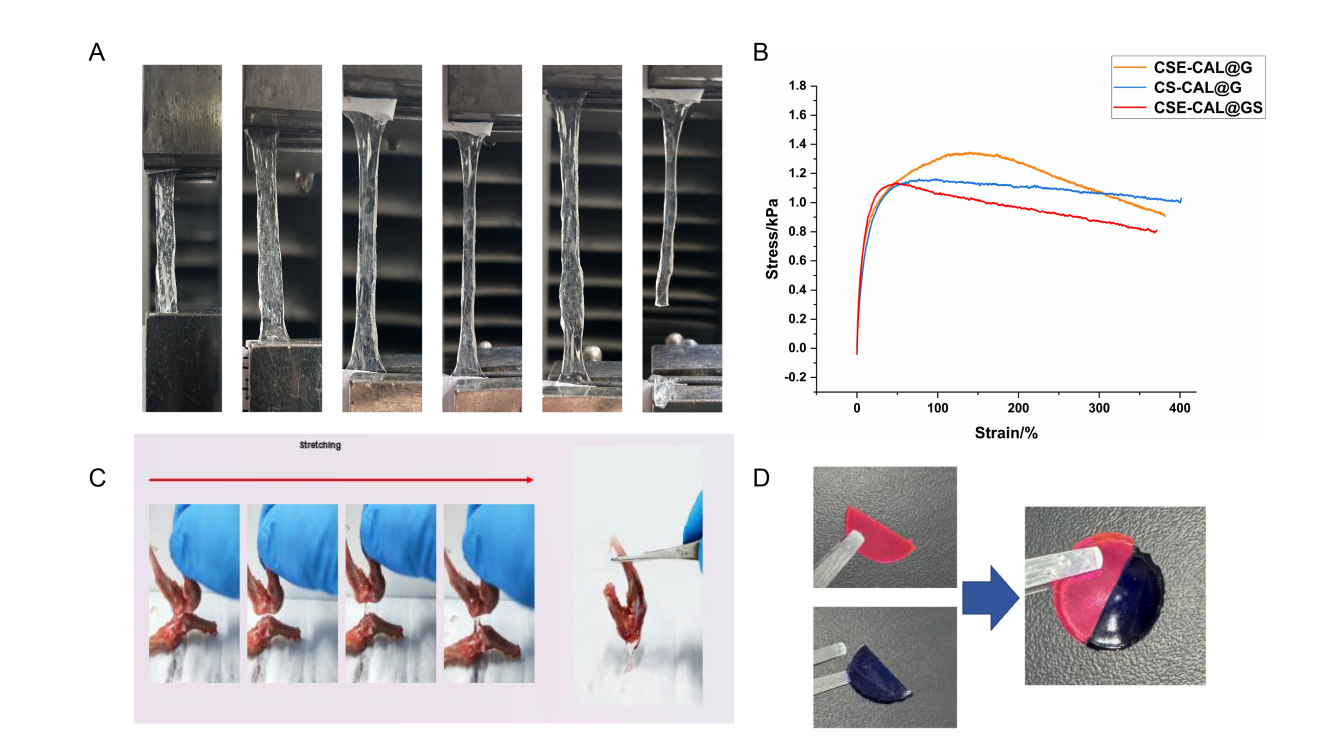
**

**Figure S5**. (A) Sequential images of hydrogel stretching between two rigid substrates during tensile testing. (B) Tensile stress-strain curves of hydrogels with different formulations (CSE-CAL@G, CS-CAL@G, and CSE-CAL@GS), showing their mechanical strength and elongation at break. (C) Adhesion test of hydrogels. (D) Images of self-healing behavior of composite hydrogel. The divided hydrogel pieces healed into a piece of hydrogel after contacting for 25 min.

**Figure S6**


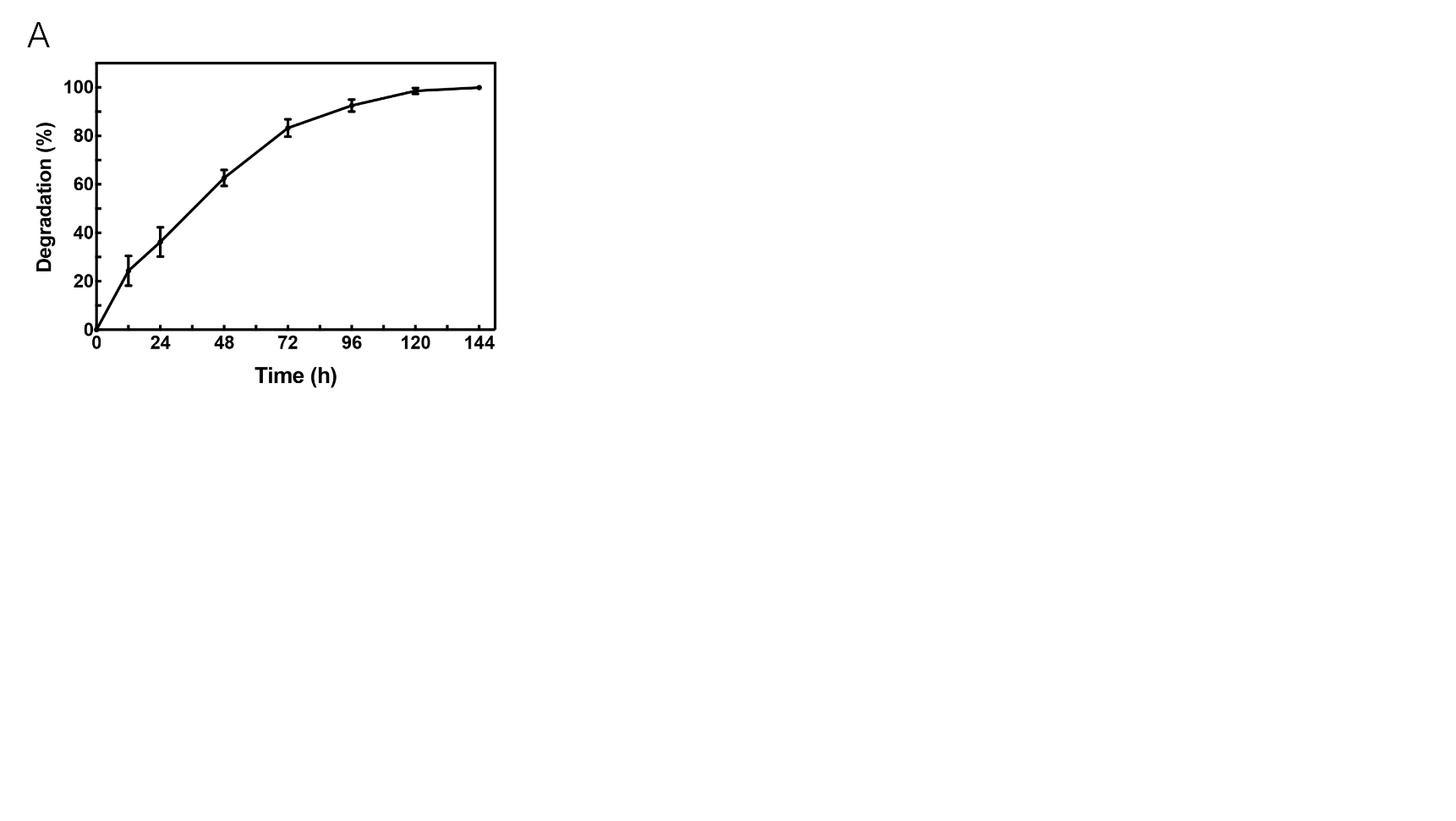


**Figure S6**. Degradation ratio of the Gelma-SDF-1α hydrogel.

**Figure S7**

**
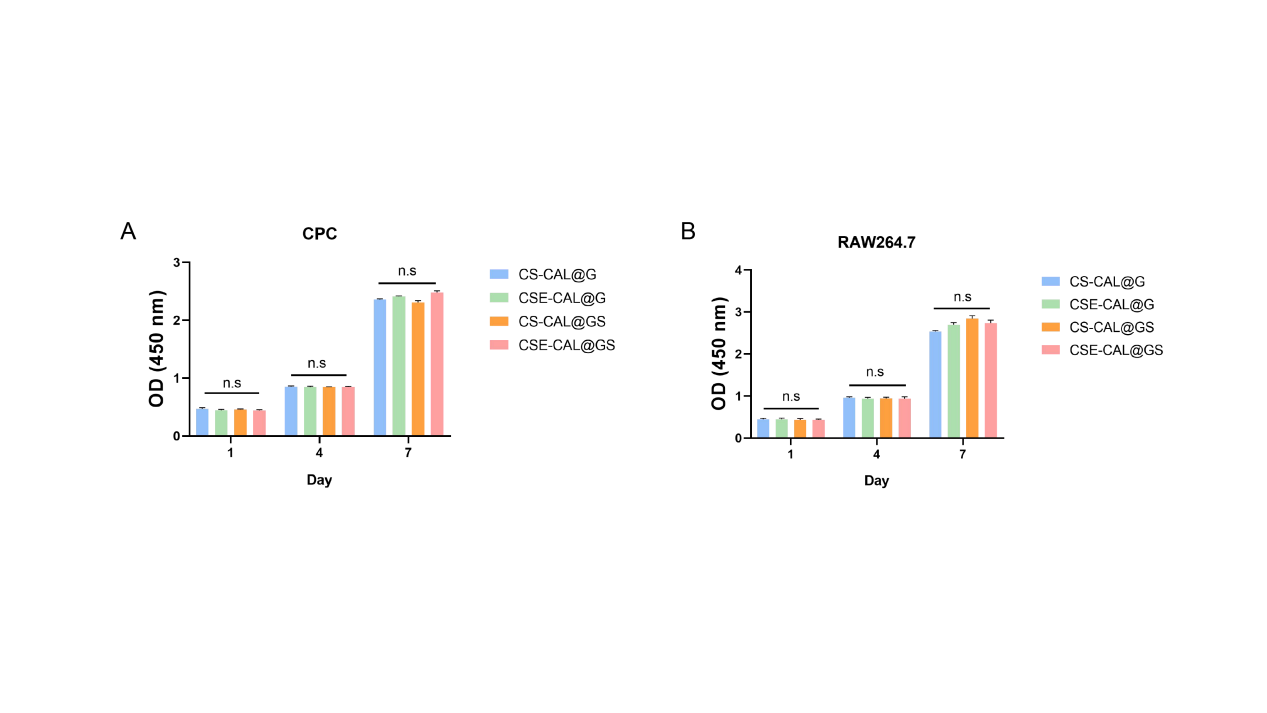
**

**Figure S7**. CCK-8 assay of cell viability in different groups. (A-B) Cell viability of precursor (pre-gel) solutions in CPCs and RAW264.7 cells. N.S., not significant.

**Figure S8**

**
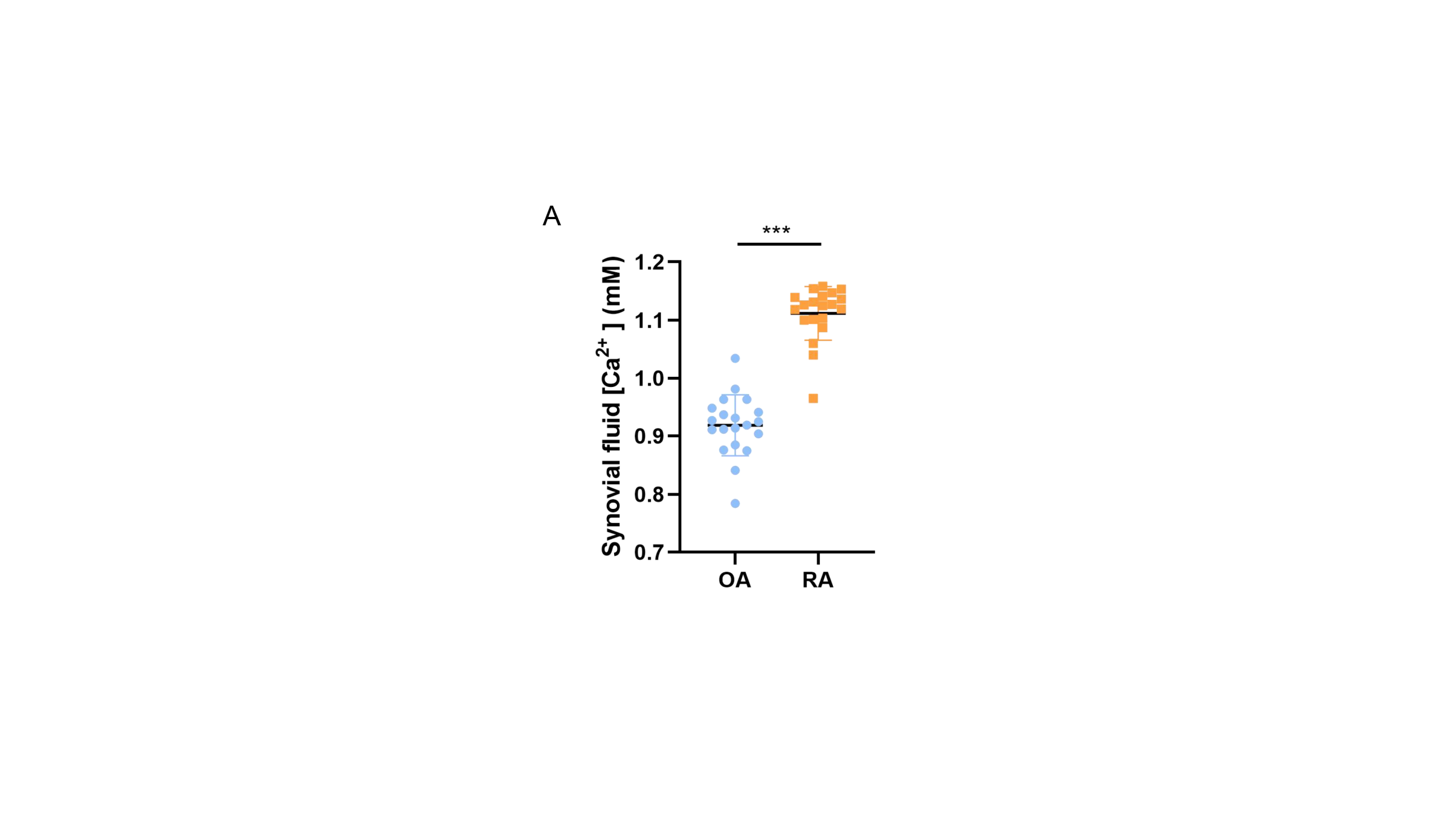
**

**Figure S8**. Measurement of Ca^2+^ in synovial fluid of either RA or control patients with OA. ****P*<0.001.

**Figure S9**

**
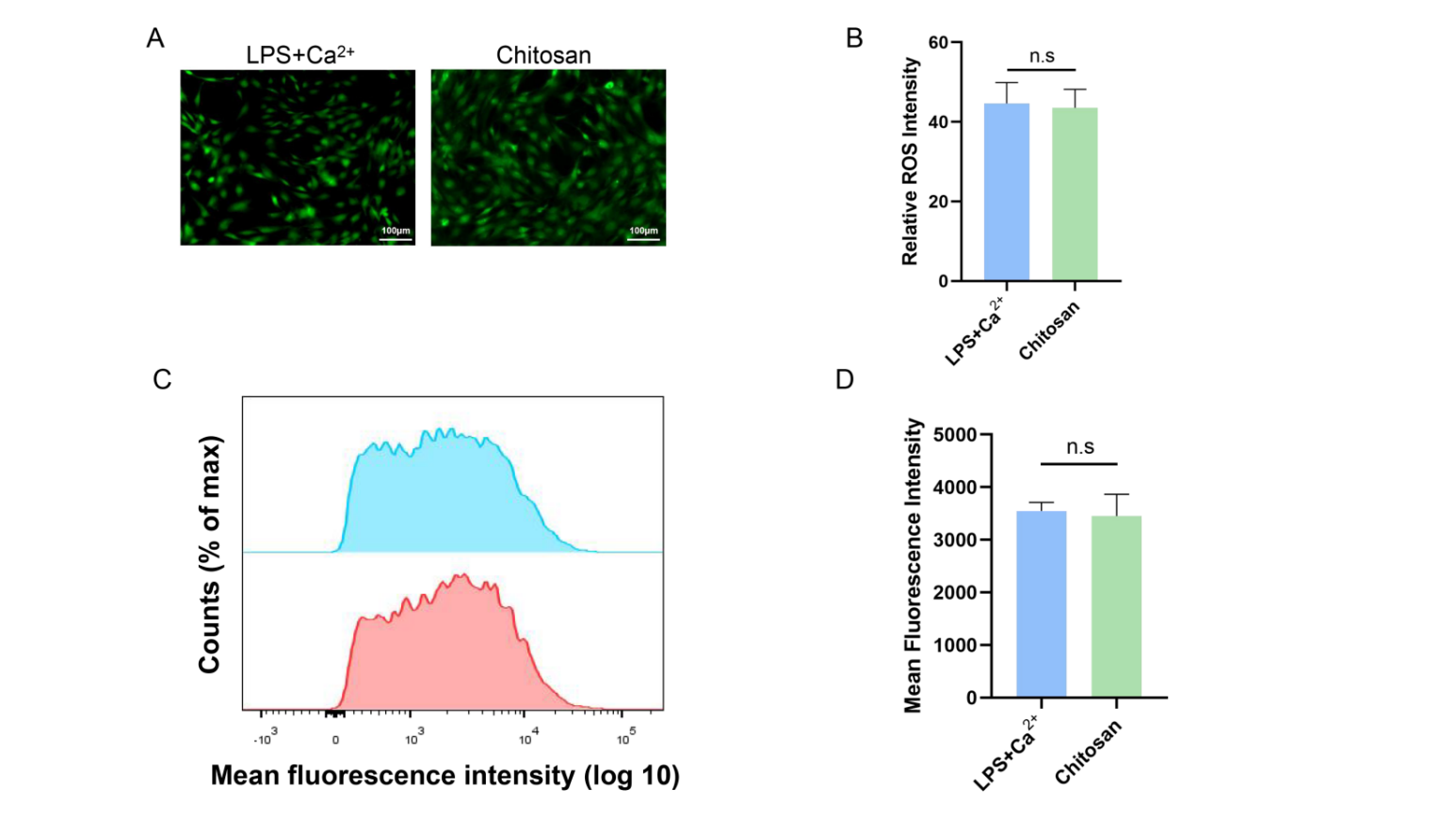
**

**Figure S9**. Chitosan does not alter intracellular ROS production in CPCs treated with LPS+Ca²⁺. (A-B) Representative fluorescence microscopy images showing DCF fluorescence (green) in cells treated with LPS + Ca²⁺ or Chitosan. (C-D) H2DCFH-DA as an intracellular ROS indicator and measured by FCM. N.S., not significant

**Figure S10**

**
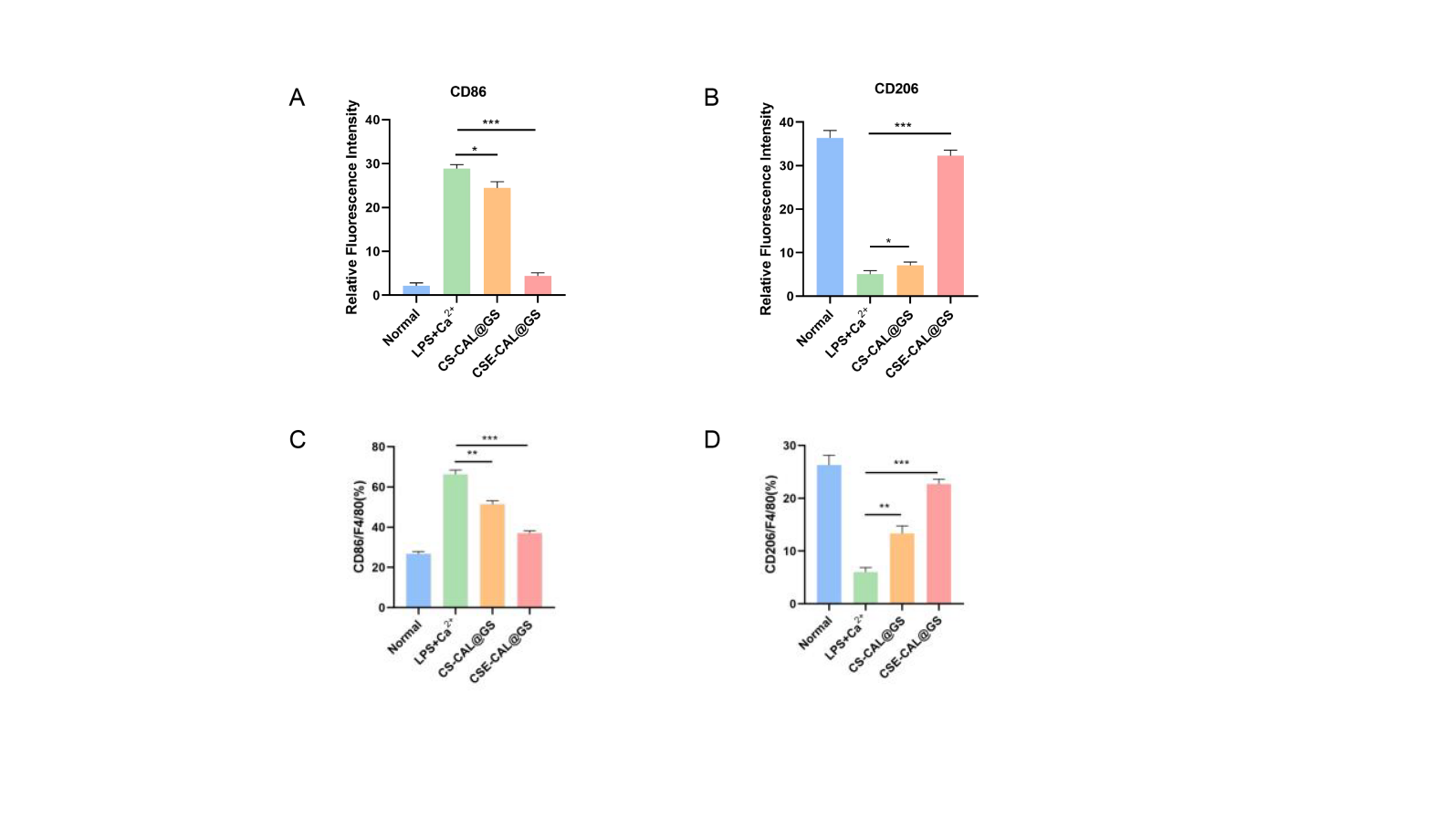
**

**Figure S10**. Quantitative validation of macrophage polarization by immunofluorescence MFI and flow cytometry in RAW264.7 cells treated with LPS+Ca²⁺. (A-B) Quantification of mean fluorescence intensity of CD86^+^ (M1 type) and CD206^+^ (M2 type). (C-D) Quantification of CD86^+^ (M1 type) and CD206^+^ (M2 type) macrophages. **P*< 0.05, ***P*<0.01, and ****P*<0.001.

**Figure S11**

**
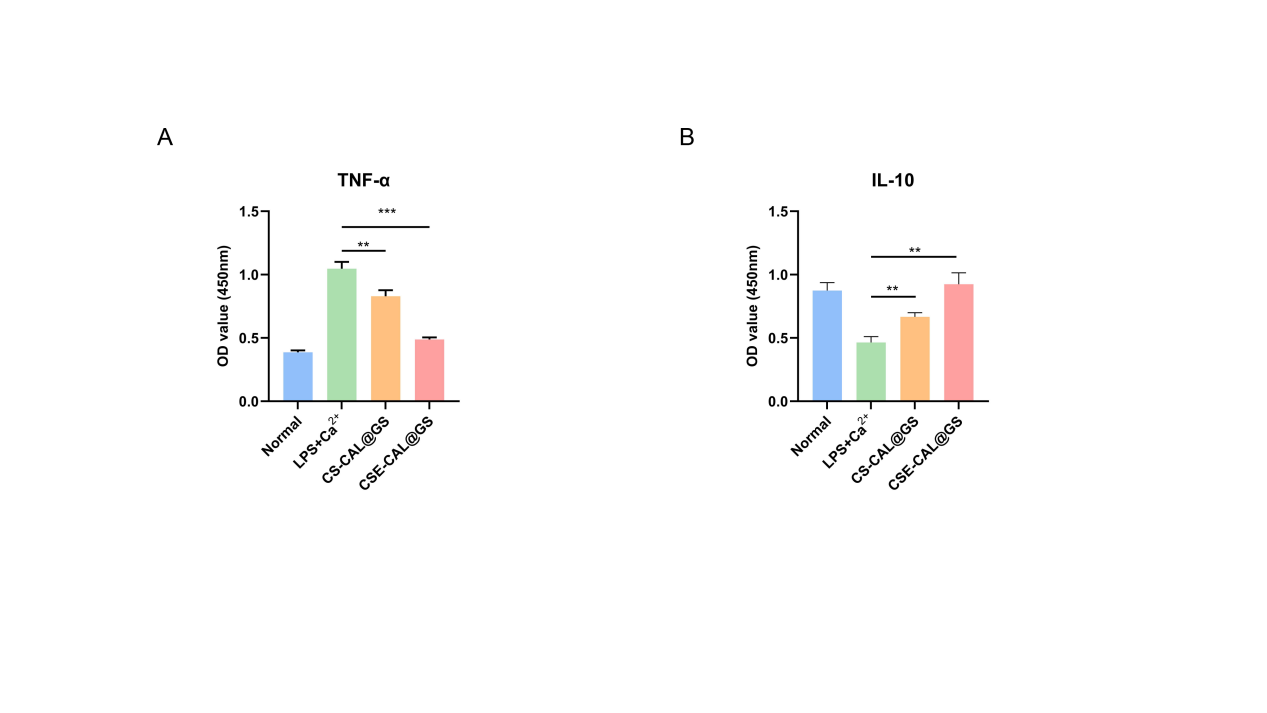
**

**Figure S11**. Quantification of cytokine secretion by RAW264.7 macrophages. (A) TNF-α levels measured by ELISA in different treatment groups. (B) IL-10 levels measured by ELISA in corresponding groups. ***P*<0.01, and ****P*<0.001.

**Figure S12**

**
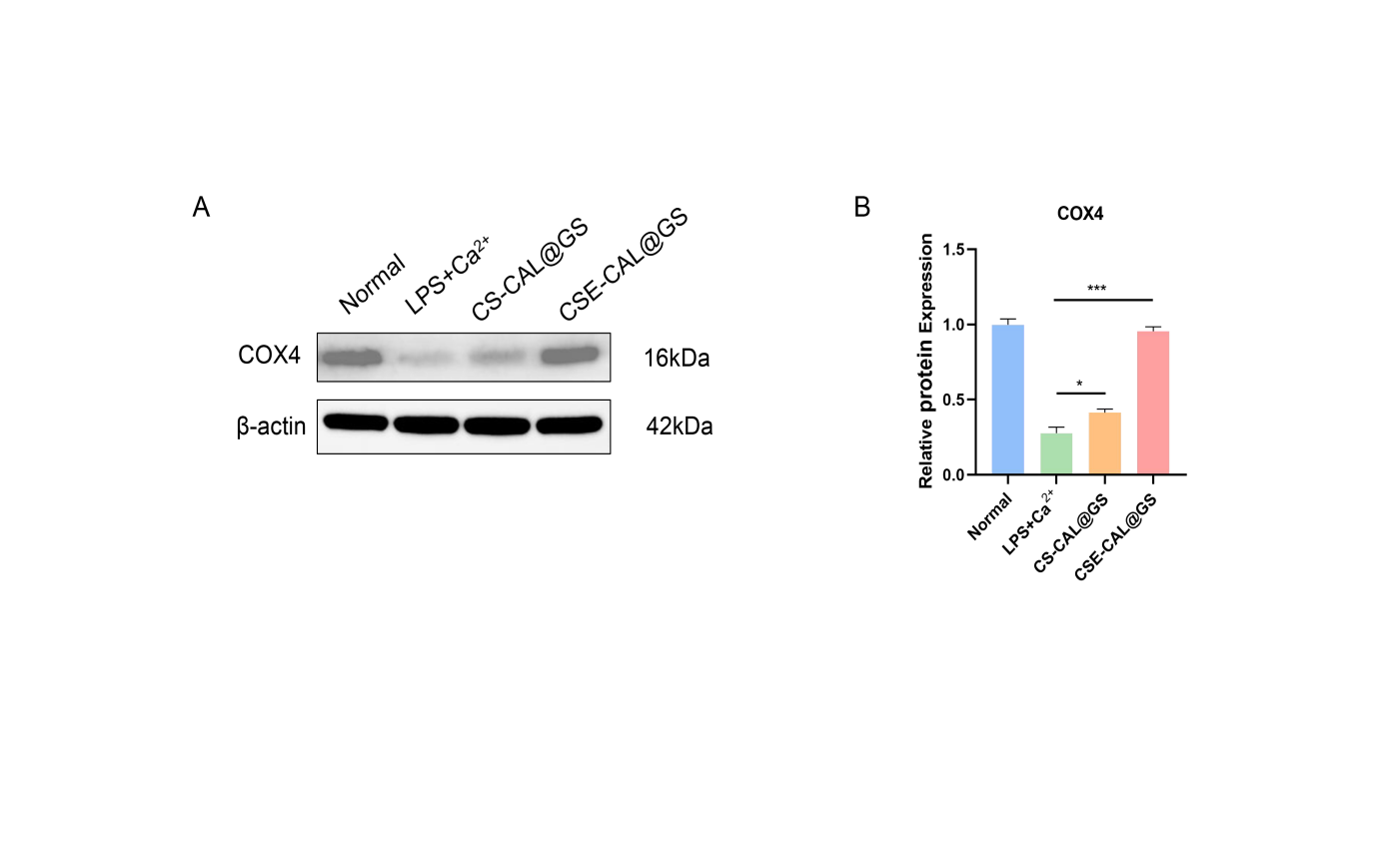
**

**Figure S12.** Western blot analysis of COX4 expression in CPCs. (A) Representative immunoblot bands of COX4 protein in different treatment groups, with β-actin used as the loading control. (B) Quantification of COX4 protein levels normalized to β-actin. *P< 0.05 and ***P<0.001.

**Figure S13**

**
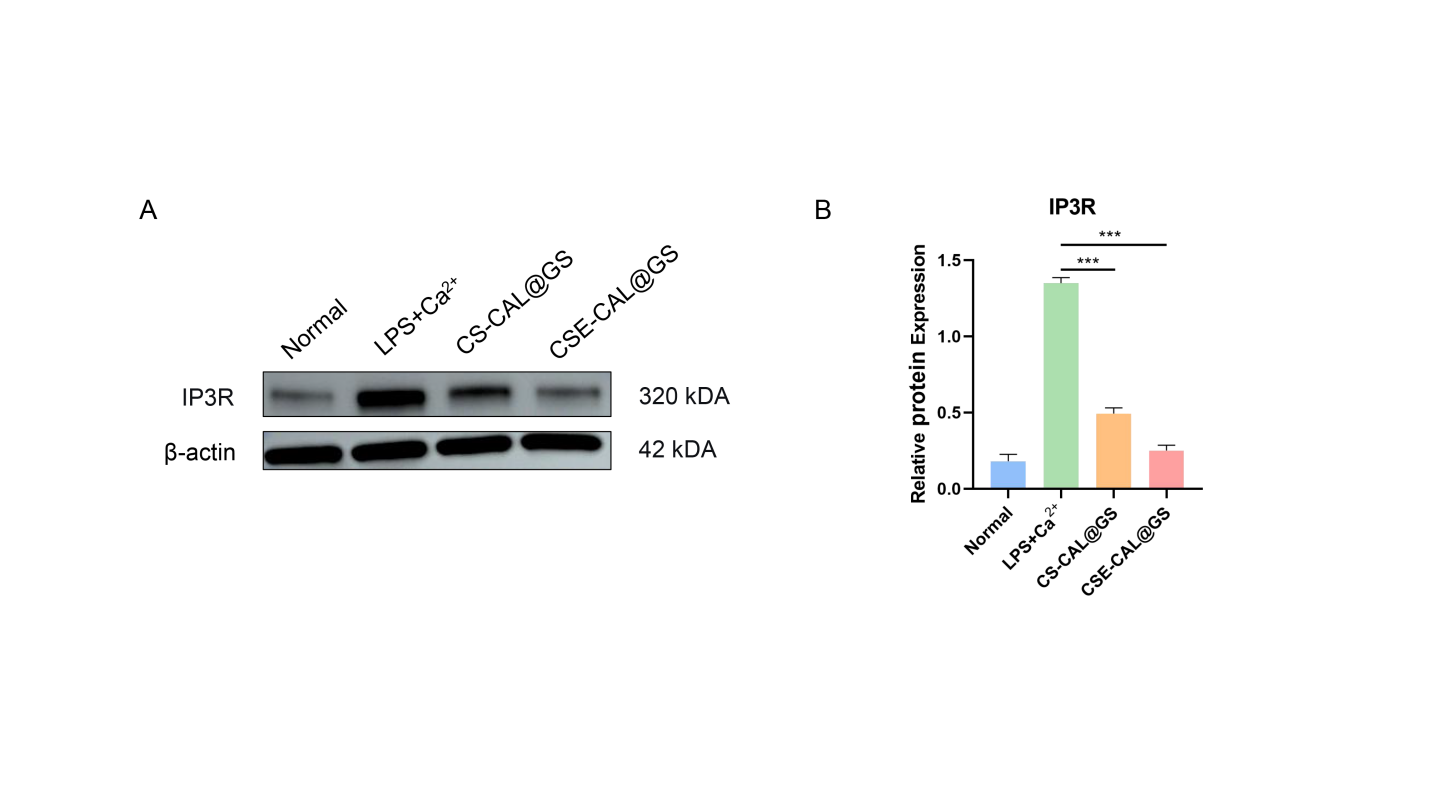
**

**Figure S13.** Western blot analysis of IP3R expression in CPCs. (A) Representative immunoblot bands of IP3R protein in different treatment groups, with β-actin used as the loading control. (B) Quantification of IP3R protein levels normalized to β-actin. ****P*<0.001.

**Figure S14**


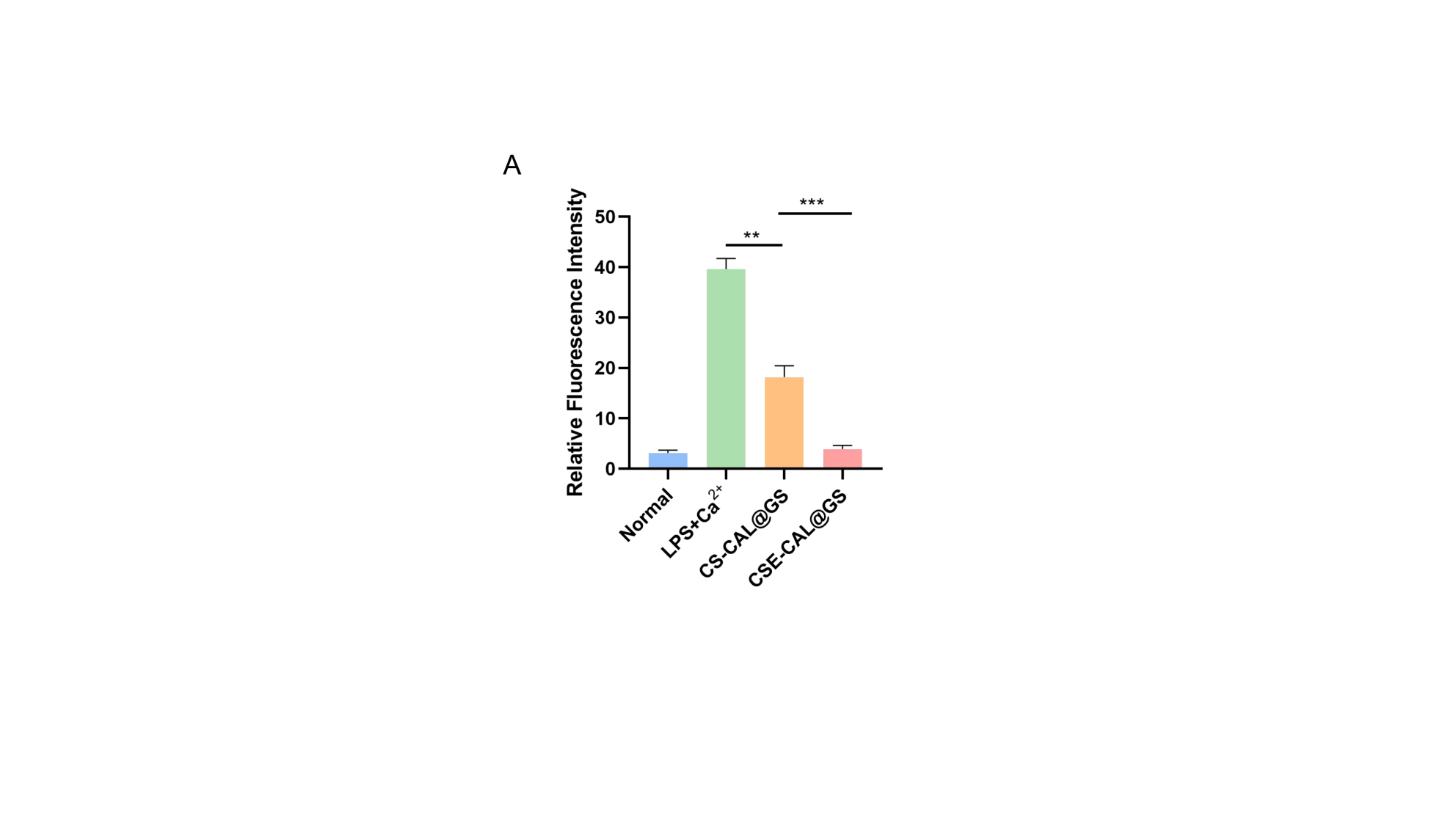


**Figure S14.** Quantification of Rhod-2 mean fluorescence intensity. ***P*<0.01, and ****P*<0.001.

**Figure S15**

**
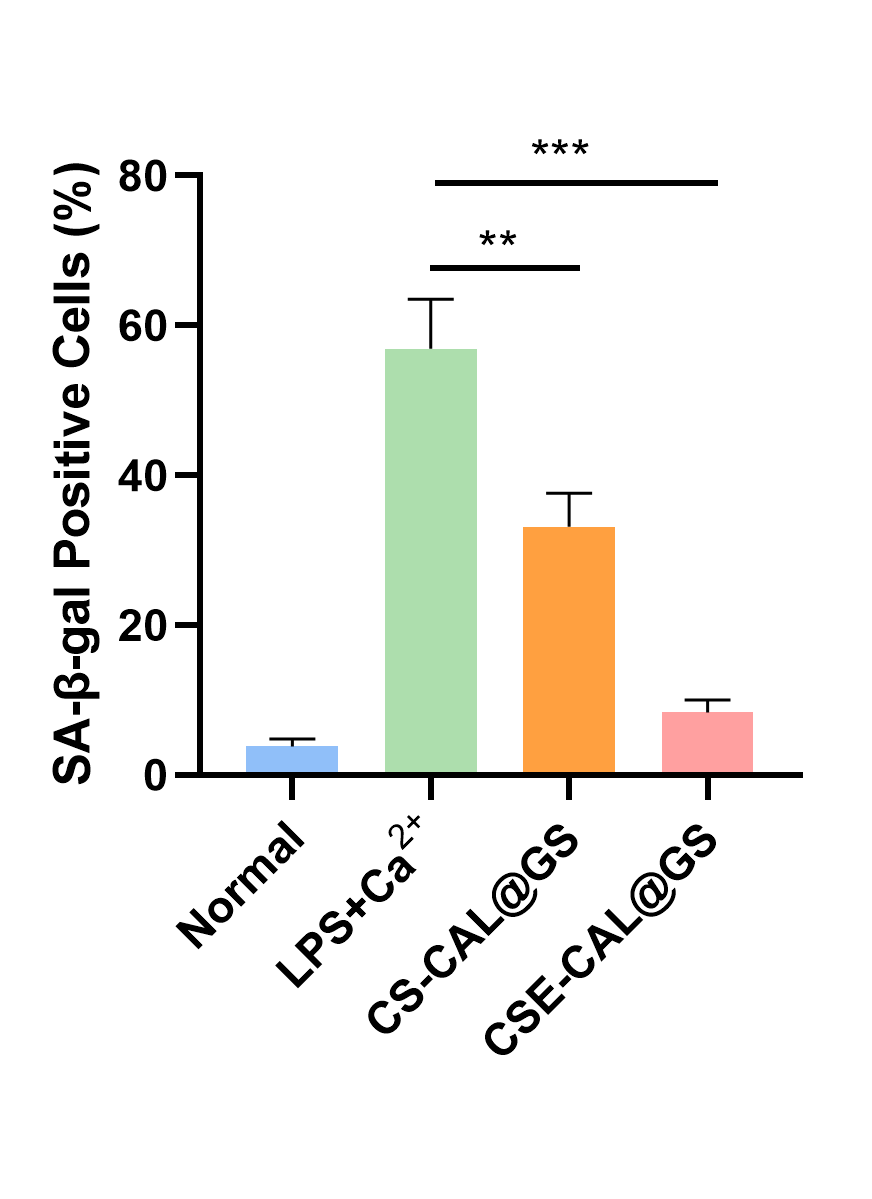
**

**Figure S15.** Quantification of senescence-associated β-galactosidase (SA-β-gal) activity in the four experimental groups. ***P*<0.01, and ****P*<0.001.

**Figure S16**

**
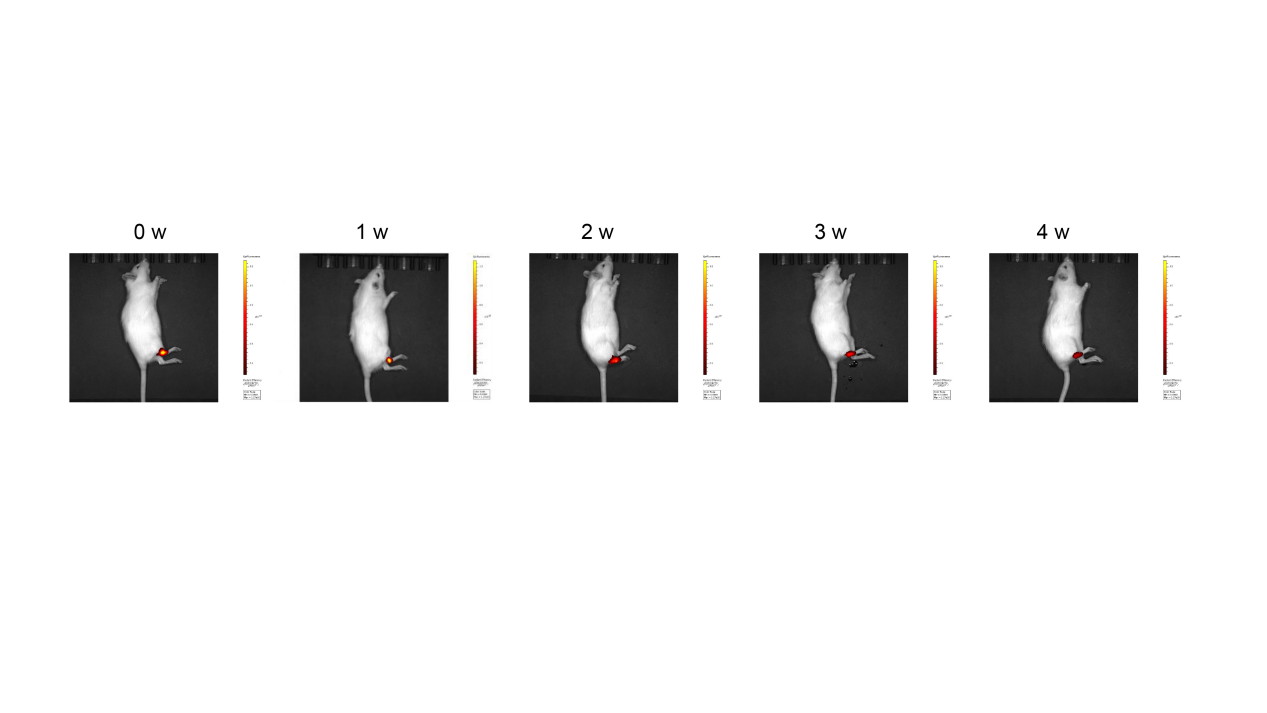
**

**Figure S16.** *In vivo* imaging system (IVIS) images of visualized CSE-CAL@GS in the articular cavity of rat at different time points.
